# Subtly Manipulated Expression of ZmmiR156 in Tobacco Improves Drought and Salt Tolerance Without Changing the Architecture of Transgenic Plants

**DOI:** 10.3389/fpls.2019.01664

**Published:** 2020-01-10

**Authors:** Tao Kang, Chun-Yan Yu, Yue Liu, Wei-Meng Song, Yan Bao, Xiao-Tong Guo, Bei Li, Hong-Xia Zhang

**Affiliations:** ^1^ College of Agriculture, Ludong University, Yantai, China; ^2^ Institute of Plant Physiology and Ecology, Shanghai Institutes for Biological Sciences, Chinese Academy of Sciences, Shanghai, China; ^3^ University of Chinese Academy of Sciences, Beijing, China; ^4^ Key Laboratory of Molecular Module-Based Breeding of High Yield and Abiotic Resistant Plants in Universities of Shandong (Ludong University), Yantai, China; ^5^ College of Life Sciences, Qingdao University, Qingdao, China

**Keywords:** drought, salt, ZmmiR156, SPL, transgenic plants

## Abstract

Plants in the juvenile state are more tolerant to adverse conditions. Constitutive expression of MicroRNA156 (miR156) prolonged the juvenile phase and increased resistance to abiotic stress, but also affected the architecture of transgenic plants. In this study, we investigated the possibility of subtle manipulation of miR156 expression in flowering plants, with the goal to increase tolerance to abiotic stress without altering the normal growth and development of transgenic plants. Transgenic tobacco plants expressing ZmmiR156 from maize were generated, driven either by the cauliflower mosaic virus (CaMV) 35S promoter or the stress-inducible ZmRab17 promoter. Expression of ZmmiR156 led to improved drought and salt tolerance in both *35S::MIR156* and *Rab17::MIR156* transgenic plants, as shown by more vigorous growth, greater biomass production and higher antioxidant enzyme expression after a long period of drought or salt treatment, when compared to wild type and transgenic vector control plants. However, constitutive expression of ZmmiR156 also resulted in retarded growth, increased branching and delayed flowering of transgenic plants. These undesirable developmental changes could be mitigated by using the stress-inducible ZmRab17 promoter. Furthermore, under drought or salt stress conditions, expression of ZmmiR156 reduced the transcript level of *NtSPL2* and *NtSPL9*, the genes potentially targeted by ZmmiR156, as well as that of *CP1*, *CP2*, and *SAG12*, the senescence-associated genes in tobacco. Collectively, our results indicate that ZmmiR156 can be temporally manipulated for the genetic improvement of plants resistant to various abiotic stresses.

## Introduction

Unlike animals, plants are sessile organisms that cannot avoid or flee from adverse conditions. Therefore, they have developed a series of strategies to cope with or tolerate different environmental stresses. Drought and salt are two of the most serious environmental factors which have severely restricted the production of agricultural crops worldwide (Rivero et al., 2007). To date, a number of drought and salt stress-related genes and pathways have been isolated and identified in different plant species (Zhang and Blumwald, 2001; Xiong et al., 2002; Yamaguchi-Shinozaki and Shinozaki, 2006; Yang et al., 2008).

In higher plants, MicroRNA156 (miR156), a class of 20–24 nucleotide non-coding regulatory RNAs derived from hairpin precursors by the Dicer-like enzymes, plays crucial roles in transcriptional and post-transcriptional gene regulation *via* the silencing mechanism (Carrington and Ambros, 2003; Bartel, 2004; Baulcombe, 2004; He and Hannon, 2004). As one of the conserved miRNA families in higher plants, miR156 is highly expressed in seedlings and decreased during the juvenile-to-adult transition (Wang et al., 2011). Together with its target *SQUAMOSA-PROMOTER BINDING PROTEIN-LIKE* (*SPL*) family, miR156 has acted as a versatile toolbox in regulating various developmental processes of plants (Wang and Wang, 2015). In *Arabidopsis* and poplar, the miRNA156/157 recognition element in the 3′ UTR of *SPL3* prevented early flowering by translational inhibition in seedlings (Gandikota et al., 2007). The miRNA regulation cascades (miR156-*SPL13*-miR172-*SNZ*) also played a crucial role during the post-germination developmental stages (Martin et al., 2010). Overexpression of miR156 delayed the phase transitions from juvenile to adult and adult to reproductive, and caused shortened plastochron and leaf overproduction in transgenic plants (Wu and Poethig, 2006; Schwarz et al., 2008; Wang et al., 2008). In maize, enhanced expression of miR156b/c dramatically increased the branch numbers of transgenic plants (Chuck et al., 2007). In rice transgenic expression of miR156 enhanced branch/tiller numbers of transgenic plants (Jiao et al., 2010). In poplar, overexpression of miR156 reduced the expression of miR156-targeted *SPL* genes, and prolonged the juvenile phase of transgenic poplar (Wang et al., 2011). Recently, overexpression of GmmiR156b in soybean improved shoot architecture and significantly increased the numbers of long branches, nodes and pods, leading to improved grain yield in transgenic plants (Sun et al., 2019).

In addition to its developmental functions, miRNAs also play crucial roles in plant response to environmental stress. In *Arabidopsis*, miR156 isoforms were induced by heat stress (HS) and promoted the expression of HS-responsive genes (Stief et al., 2014). In barley, Hvu-miR156, Hvu-miR166, Hvu-miR171, and Hvu-miR408 were responsive to drought stress (Kantar et al., 2010). In maize, miRNA399b, miRNA156, and Zma-miR3 were induced by low phosphorus stress (Zhang et al., 2012). Transgenic *Arabidopsis* plants expressing soybean GmmiR172c exhibited increased resistance to drought and salt stresses (Li et al., 2016). Expression of miRNA156 in *Arabidopsis* and rice led to improved tolerance to NaCl and osmotic stresses in transgenic plants (Cui et al., 2014). In alfalfa, expression of miR156 reduced plant height and increased biomass, branch number and time to complete vegetative growth under both normal and salt stress conditions (Arshad et al., 2017). Similarly, in chickpea, overexpression of miR408 led to reduced plant height and increased drought tolerance (Hajyzadeh et al., 2015).

In this study, we explored whether subtly manipulating the expression of ZmmiR156 from maize (*Zea mays*) in tobacco would improve the tolerance to drought and salt stresses but cause no significant effects on the regular growth and development of transgenic plants. Our results demonstrate that, by temporally regulating the expression of ZmmiR156 with a stress-inducible promoter, transgenic plants resistant to both drought and salt stresses with no significant morphological changes can be generated.

## Materials and Methods

### Plant Materials and RNA Isolation

Tobacco plants (*Nicotiana tabacum* cv. *xanthi*) were grown on MS medium (Murashige and Skoog, 1962) with 2% (w/v) sucrose and 0.8% (w/v) agar with cool white fluorescent light (~200 μmol/m^2^/s) under a 12 h light/12 h dark photoperiod at 21–25°C/15–18°C (day/night). Plants in soil were grown in a greenhouse under a 14 h photoperiod, comprised of natural daylight supplemented with lamps (120–150 µEm^2^/s) at 21–25°C/15–18°C (day/night). The sequenced maize (*Zea mays* L.) cultivar B73 was grown in greenhouse as described previously (Li et al., 2013).

For expression pattern analyses of ZmmiR156 in maize plants, juvenile (1-month-old) and old (4-month-old) maize plants or roots of 2-week-old seedlings treated with 15% PEG, 100 mM NaCl or 50 μM ABA were used. Total RNA was extracted from different tissues with RNAiso Plus and RNAiso-mate for Plant Tissue (TaKaRa, Dalian, China), and treated with RNase-free DNase (Promega, Shanghai, China).

### Vector Construction and Plant Transformation

The ZmmiR156 sequence was cloned from B73 (GenBank accession No. EF541486.1). The 764-bp pre-mature miR156 sequence was inserted into a modified pCAMBIA3301 vector *via* the *Bam* HI and *Spe* I restriction sites, under the control of two copies of the cauliflower mosaic virus (CaMV) 35S promoter or the drought- and ABA-inducible maize Rab17 promoter. The resultant constructs, as well as the modified pCAMBIA3301 vector, were respectively introduced into *Agrobacterium tumefaciens* strain EHA105 for tobacco transformation. Leaves of *N. tabacum* cv. *xanthi* were transformed as described previously (Yang et al., 2008). Independently regenerated transgenic shoots were propagated and transplanted into soil for homozygous seed production. Transgenic plants transformed with the modified pCAMBIA3301 vector were used as vector control.

### Sequence Alignment and Phylogenetic Analyses

Sequence alignment of mature NtmiR156 and ZmmiR156 was performed on Clustal omega website (http://www.ebi.ac.uk/Tools/msa/clustalo). Phylogenetic trees were generated using the MEGA 7.0 and drawn using the neighbor joining method. The accession numbers for *AtSPLs*, *NtSPLs*, and *ZmSPLs* are shown in **Supplementary Table S1**.

### Reverse Transcription PCR and Quantitative Real-Time PCR

First-strand cDNA was synthesized using the ReverTra Ace Kit (TOYOBO, Osaka, Japan) following the manufacturer’s instruction. A total amount of 2 µg RNA was subjected to reverse transcription reaction using ReverTra Ace (Vazyme, Shanghai, China) at 50°C for 30 min. The resultant cDNA was then used for PCR amplification with gene-specific primers. *ZmActin* and *NtActin* were employed as internal controls, respectively. Quantitative real-time PCR (qRT-PCR) was performed with the SYBR Green Real-time PCR Master Mix (Vazyme, Shanghai, China) and monitored in real time with the CFX Connect (BIO-RAD, Shanghai, China). All the primers used in this research are listed in **Supplementary Table S2**.

### Drought and Salt Stress Treatments

For drought stress analyses, 4-week-old seedlings for *35S::MIR156#1* and *35S::MIR156#2* and 6-week-old seedlings for *Rab17::MIR156#1* and *Rab17::MIR156#7* were withheld from watering for another four weeks. For salt stress treatment, 2-month-old *35S::MIR156#1* and *35S::MIR156#2* and 6-week-old *Rab17::MIR156#1* and *Rab17::MIR156#7* plants grown in greenhouse were watered with 1/8 concentration of MS salt solution supplemented with or without 200 mM NaCl bi-weekly for 6 weeks.

### Determination of Malondialdehyde (MDA) and Proline Concentration

To measure the content of MDA, leaf samples were ground in 5 ml of 0.1% trichloroacetic acid (TCA) and mixed with 5 ml of 0.5% thiobarbituric acid. The samples were then boiled for 10 min, cooled to room temperature and centrifuged at 12,000 *g*. The supernatant was analyzed by monitoring the difference in absorbance at *A_532_* and *A_600_*. Proline content was determined as described previously (Bates and Walden, 1973).

### Nitroblue Tetrazolium (NBT) Staining and Chlorophyll Fluorescence Assays

Leaf disks were stained with nitroblue tetrazolium (NBT) as described previously (Fryer et al., 2002). To measure chlorophyll fluorescence concentration, the fourth new grown leaf counted from the bottom of each plant was taken and photographed at the end of the relative treatment. Chlorophyll content was determined as described by Lichtenthaler (1987).

### Measurements of Stomatal Density and Aperture

For stoma observation, epidermal strips were peeled from the leaves of two-month-old wild type, vector control and transgenic plants. The samples were incubated in a solution containing 10 mM KCl, 10 mM MES-Tris, and 50 µM CaCl_2_ (pH 6.15), and exposed to light (100 µmol m^−2^ s^−1^) for 3 h. Subsequently, 0 µM or 50 µM ABA was added to the solution. After 2 h, samples were examined under 20× and 40× magnification using a Nikon microscope. The number of stomata was counted, and the widths and lengths of stomatal apertures were measured. Mean ratios of stomatal density and width to length ± SE of three independent experiments (n = 30–50) were calculated.

### Statistical Analysis

All the data were normalized and all samples were normally distributed with homogeneity of variance. Error bars represent the SDs from three biological replicates. Student’s t-test was used for statistical analyses. The tests were one-tailed.

## Results

### Cloning and Expression Analyses of ZmmiR156 in Maize

To understand the biological functions of miR156 in plant responses to abiotic stresses, the full length sequence of ZmmiR156 was isolated from maize. Consistent with previous reports that miR156 is highly conserved in plants, ZmmiR156 shares very high sequence identity with miR156s in tobacco (Axtell and Bowman, 2008; **Supplementary Figure S1A**). Since the miR156/*SPLs* regulatory module was conserved in diverse plant species, we also conducted phylogenetic analyses of *SPLs* from *Arabidopsis*, tobacco and maize using MEGA 7.0 (Chuck et al., 2007; Wang et al., 2011; Wang et al., 2014; **Supplementary Figure S1B**).

To see whether ZmmiR156 is responsive to abiotic stress, we performed qRT-PCR analyses. Under normal growth condition, ZmmiR156 was ubiquitously expressed in different tissues and organs including roots, leaves, and stems, with a relatively higher expression in juvenile roots and leaves (**Supplementary Figure S2A**). Upon treatments with 15% PEG, 100 mM NaCl or 50 μM ABA, ZmmiR156 transcript level increased after 1 h and then decreased after 6 h (**Supplementary Figures S2B**–**D**). These results demonstrate that ZmmiR156 is responsive to different abiotic stresses and ABA.

### Generation of Transgenic Plants Expressing ZmmiR156

To explore whether it is feasible to improve the resistance to different abiotic stresses without changing the architecture of transgenic plants, transgenic tobacco plants with constitutive or stress-induced ZmmiR156 expression were generated. ZmmiR156 sequence was cloned into the pCAMBIA-3301 vector, under the control of either two copies of the cauliflower mosaic virus 35S (2×35S) promoter or the stress-inducible ZmRab17 promoter, and introduced into the genome of tobacco (*N. tabacum* cv. *xanthi*) by *A. tumefaciens*-mediated transformation (**Supplementary Figures S3A**, **B**). Fifty-two independently regenerated transgenic lines (T_0_ generation) were obtained, and twenty lines were confirmed by PCR for the integration of ZmmiR156. The expression level of ZmmiR156 in different transgenic lines was examined by qRT-PCR. Two homozygous *35S::ZmmiR156* transgenic lines which showed high expression of ZmmiR156 (*35S::MIR156#1*, *35S::MIR156#2*) and two homozygous *Rab17::MIR156* transgenic lines which showed very low expression of ZmmiR156 (*Rab17::MIR156#1*, *Rab17::MIR156#7*) were selected for further stress resistance and morphological analyses (**Supplementary Figures S3C**, **D**).

### ZmmiR156 Expression Increased Osmotic and Salt Tolerance in Transgenic Tobacco Seedlings

**Figure 1:**
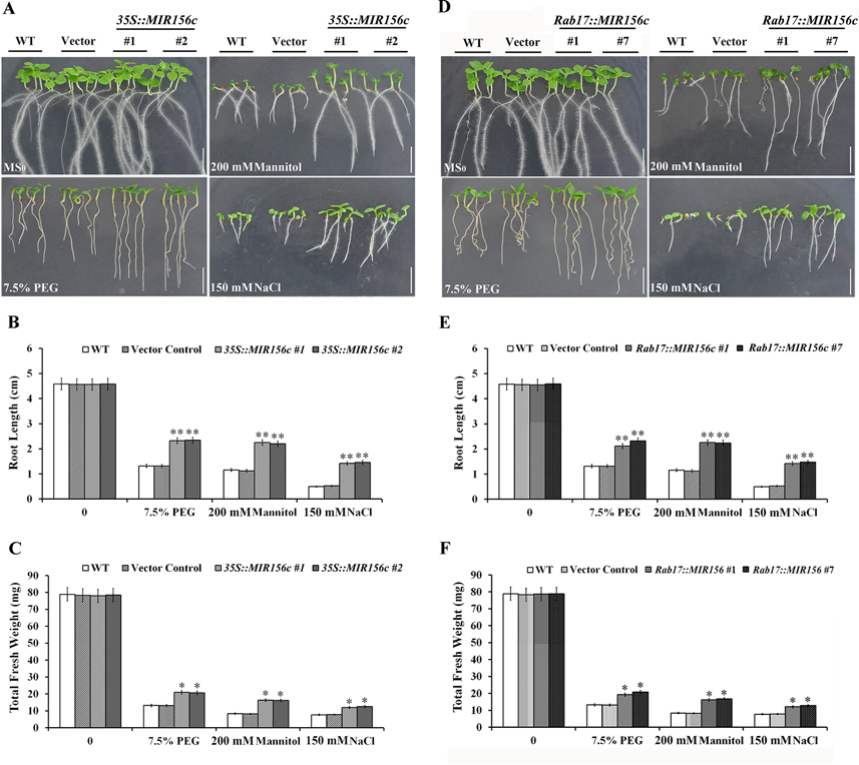
Osmotic and salt stress analyses of wild type (WT), vector control, and both *35S::MIR156,* and *Rab17::MIR156* transgenic seedlings. Seeds of WT, vector control and transgenic lines *35S::MIR156#1*, *35S::MIR156#2*, *Rab17::MIR156#1*, and *Rab17::MIR156#7* were sown on MS medium supplemented with 7.5% PEG, 200 mM mannitol, or 150 mM NaCl and vertically cultured for two weeks. **(A)** Phenotypes of WT, vector control and *35S::MIR156* transgenic seedlings. **(B**, **C)** Root lengths and fresh weights of seedlings in **(A)**. **(D)** Phenotypes of WT, vector control and *Rab17::MIR156* transgenic seedlings. **(E**, **F)** Root lengths and fresh weights of seedlings in **(D)**. Data are shown as mean ± SD from three biological replicates. Asterisks indicate significant differences from the corresponding control values at *0.01 < P < 0.05 and **P < 0.01. Scale bar = 1 cm.

We first assessed the resistance of ZmmiR156-expressing plants to osmotic or salt stress at the early developing stages of seedlings. Seeds of wild type, vector control and the four transgenic lines *35S::MIR156#1*, *35S::MIR156#2*, *Rab17::MIR156#1* and *Rab17::MIR156#7* were sown on MS medium supplemented with 7.5% PEG, 200 mM mannitol or 150 mM NaCl. The growth of wild type, vector control and transgenic seedlings were approximately the same when cultured on normal MS medium (**Figures 1A–F**). However, in the presence of 7.5% PEG, 200 mM mannitol or 150 mM NaCl, growth of wild type, vector control and transgenic seedlings was all impaired, although the growth of transgenic seedlings was less severely affected (**Figures 1A–F**). Transgenic seedlings produced longer roots and greater biomass than the wild type and vector control seedlings (**Figures 1A–F**). These results indicate that the expression of ZmmiR156 increased the osmotic and salt tolerance in tobacco.

### ZmmiR156 Expression Improved Drought and Salt Tolerance in Transgenic Adult Plants

**Figure 2:**
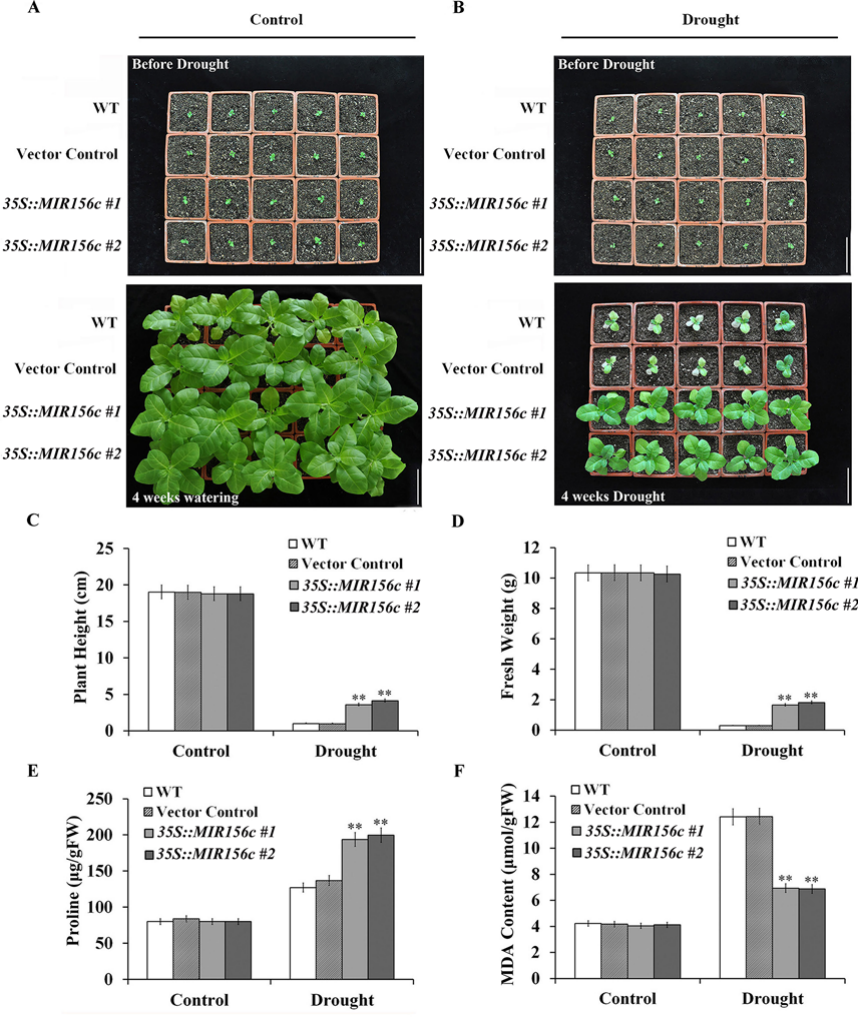
Drought tolerance, plant heights, fresh weights, and leaf proline and MDA content analyses of wild type (WT), vector control and *35S::MIR156* transgenic plants grown in greenhouse. Four-week-old seedlings of WT, vector control, and transgenic lines *35S::MIR156#1* and *35S::MIR156#2* were withheld from watering for four weeks. **(A)** Phenotypes of well-watered seedlings and plants at the beginning and end of experiment, respectively. **(B)** Phenotypes of drought-treated seedlings and plants at the beginning and end of experiment, respectively. **(C**–**F)** Plant heights, fresh weights, and leaf proline and MDA contents of plants in **(A)** and **(B)** at the end of experiment. Data are shown as mean ± SD from three biological replicates. Asterisks indicate significant differences from the corresponding control values at **P < 0.01. Scale bar = 10 cm.

**Figure 3:**
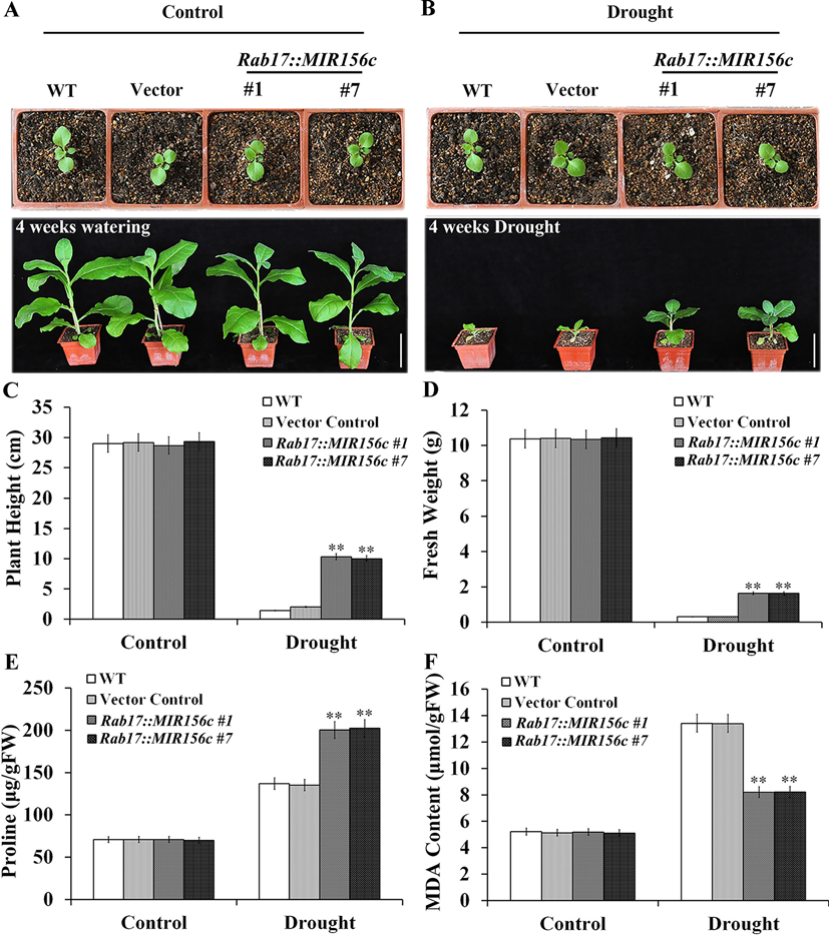
Drought tolerance, plant heights, fresh weights, and leaf proline and MDA content analyses of wild type (WT), vector control and *Rab17::MIR156* transgenic plants grown in greenhouse. Six-week-old seedlings of WT, vector control, and transgenic lines *Rab17::MIR156#1* and *Rab17::MIR156#7* were withheld from watering for four weeks. **(A)** Phenotypes of well-watered seedlings and plants at the beginning and end of experiment, respectively. **(B)** Phenotypes of drought treated seedlings and plants at the beginning and end of experiment, respectively. **(C**–**F)** Plant heights, fresh weights, and leaf proline and MDA contents of plants in **(A)** and **(B)** at the end of experiment. Data are shown as mean ± SD from three replicates. Asterisks indicate significant differences from the corresponding control values at **P < 0.01. Scale bar = 10 cm.

To further test the effect of drought on the growth of wild type and ZmmiR156-expressing plants, seedlings grown in soil in greenhouse were withheld from watering for 4 weeks. To minimize the growth retardation caused by the constitutive expression of ZmmiR156, 4-week-old seedlings of *35S::MIR156#1* and *35S::MIR156#2*, instead of 6-week-old seedlings of *Rab17::MIR156#1* and *Rab17::MIR156#7*, were used for drought treatment. Expression of ZmmiR156 did not cause any significant morphological changes since wild type, vector control, and both *35S::ZmmiR156* and *Rab17::MIR156* transgenic plants all grow normally under well-watered condition (**Figures 2A** and **3A**). After 4 weeks of water withholding, the growth of wild type and vector control plants was dramatically retarded, whereas the growth of both *35S::ZmmiR156* and *Rab17::MIR156* transgenic plants was less severely retarded (**Figures 2B** and **3B**). The plant heights and fresh weights of transgenic plants were remarkably higher compared to the wild type and vector control plants (**Figures 2C, D**, and **3C, D**).

**Figure 4:**
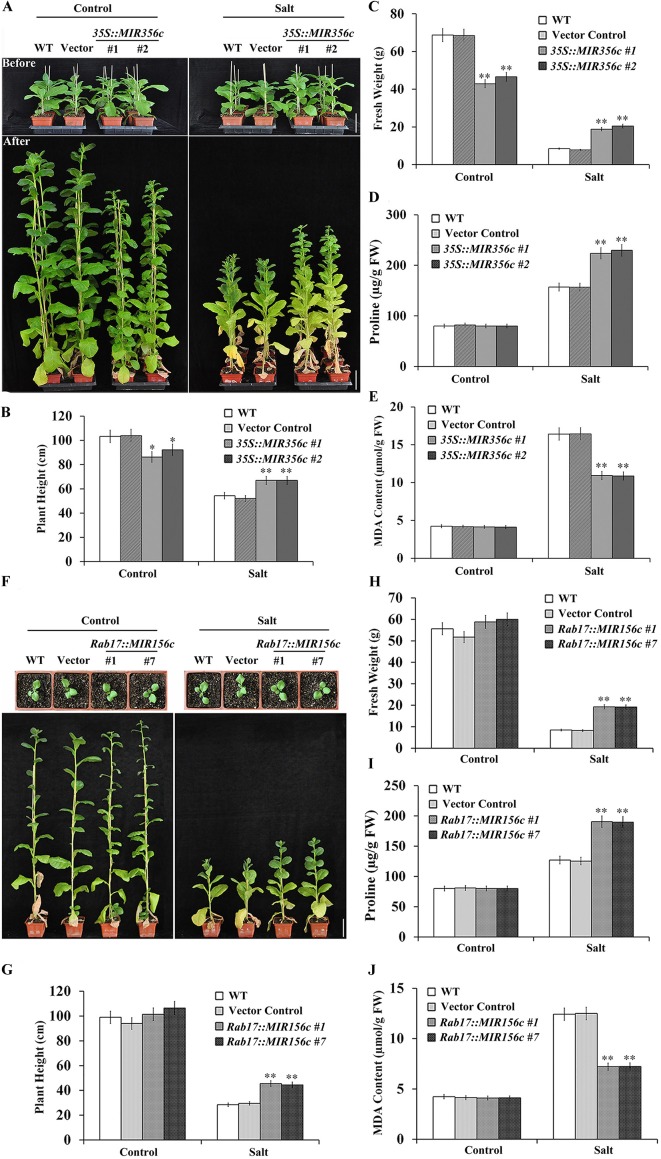
Salt tolerance, plant heights, fresh weights, leaf proline and MDA content analyses of wild type (WT), vector control, and both *35S::MIR156* and *Rab17::MIR156* transgenic plants grown in greenhouse. **(A)** Phenotypes of plants before and after the salt treatment. Two-month-old plants of WT, vector control, and transgenic lines *35S::MIR156#1* and *35S::MIR156#2* were treated with 200 mM NaCl for six weeks. **(B**–**E)** Plant heights, fresh weights, and leaf proline and MDA content of plants in **(A)** at the end of experiment. **(F)** Phenotypes of plants before and after the salt treatment. Six-week-old plants of WT, vector control, and transgenic lines *Rab17::MIR156#1* and *Rab17::MIR156#7* were treated with 200 mM NaCl for six weeks. **(G**–**J)** Plant heights, fresh weights, and leaf proline and MDA content of plants in **(F)** at the end of experiment. Data are shown as mean ± SD from three biological replicates. Asterisks indicate significant differences from the corresponding control values at **P < 0.01. Scale bar = 10 cm.

Since miR156 plays a pivotal role in regulating the transition of plants from vegetative growth to reproductive phase, we further examined the effect of salt stress on the growth of adult plants expressing ZmmiR156. Two-month-old transgenic lines *35S::MIR156#1* and *35S::MIR156#2* plants or 6-week-old transgenic lines *Rab17::MIR156#1* and *Rab17::MIR156#7* seedlings grown in greenhouse were watered with 1/8 concentration of MS salt solution supplemented with or without 200 mM NaCl bi-weekly for 6 weeks. Under normal growth condition, constitutive expression of ZmmiR156 led to retarded growth, promoted branching and delayed flowering in *35S::MIR156* transgenic plants (**Figure 4A**; **Supplementary Figures S4A**, **B**, and **S5A**, **B**). The plant heights and fresh weights of transgenic lines *35S::MIR156#1* and *35S::MIR156#2* were drastically lower than those of the wild type and vector control plants (**Figures 4B, C**). However, stress-induced expression of ZmmiR156 only caused very minor growth changes in *Rab17::MIR156* transgenic plants (**Figure 4F**; **Supplementary Figures S4A**, **B**, and **S5A**, **B**). The plant heights and fresh weights of transgenic lines *Rab17::MIR156#1* and *Rab17::MIR156#7* were approximately the same as those of the wild type and vector control plants, although flowering time was slightly delayed (**Figures 4G, H**; **Supplementary Figures S4A**, **B**, and **S5A**, **B**). Upon treatment with 200 mM NaCl, both *35S::ZmmiR156* and *Rab17::MIR156* transgenic plants showed improved tolerance to salt stress (**Figures 4A, F**). The plant heights and fresh weights of transgenic lines *35S::MIR156#1*, *35S::MIR156#2*, *Rab17::MIR156#1* and *Rab17::MIR156#7* were significantly higher than those of the wild type and relative vector control plants (**Figures 4B, C, G, H**).

### ZmmiR156 Expression Increased Oxidative Stress Tolerance in Transgenic Tobacco Plants

Since drought and salt stresses also exert osmotic stress to plant cells, we further explored whether expression of ZmmiR156 confers elevated resistance to oxidative stress on transgenic plants. It is well known that proline accumulates in response to drought or salt stress in both prokaryotic and eukaryotic organisms (Schobert 1997,). We examined the proline content in the leaves of wild type, vector control and transgenic plants grown under both normal and stress conditions. We observed that although proline accumulation increased in all plants upon the treatment of drought or salt stress, proline concentration was significantly higher in transgenic plants compared to that in the wild type and vector control plants (**Figures 2E**, **3E**, and **4D, I**).

Lipid hydroperoxidation is used as an indicator of cell damage caused by oxidative stress (Yoshimura et al., 2004). We measured the lipid hydroperoxide production rate changes induced by oxidative stress in the leaves of wild type, vector control and transgenic plants grown under both normal and stress conditions by determining the content of MDA. Under either drought or salt stress condition, MDA content increased in all plants, but was prominently lower in transgenic plants than that in wild type and vector control plants (**Figures 2F**, **3F**, and **4E, J**). Although the expression of ascorbate peroxidase (APX), catalase (CAT) and superoxide dismutase (SOD) was approximately the same under normal conditions, a higher expression was observed in transgenic plants under both drought and salt stress conditions (**Supplementary Figures S6A**–**F** and **S7A**–**F**).

**Figure 5:**
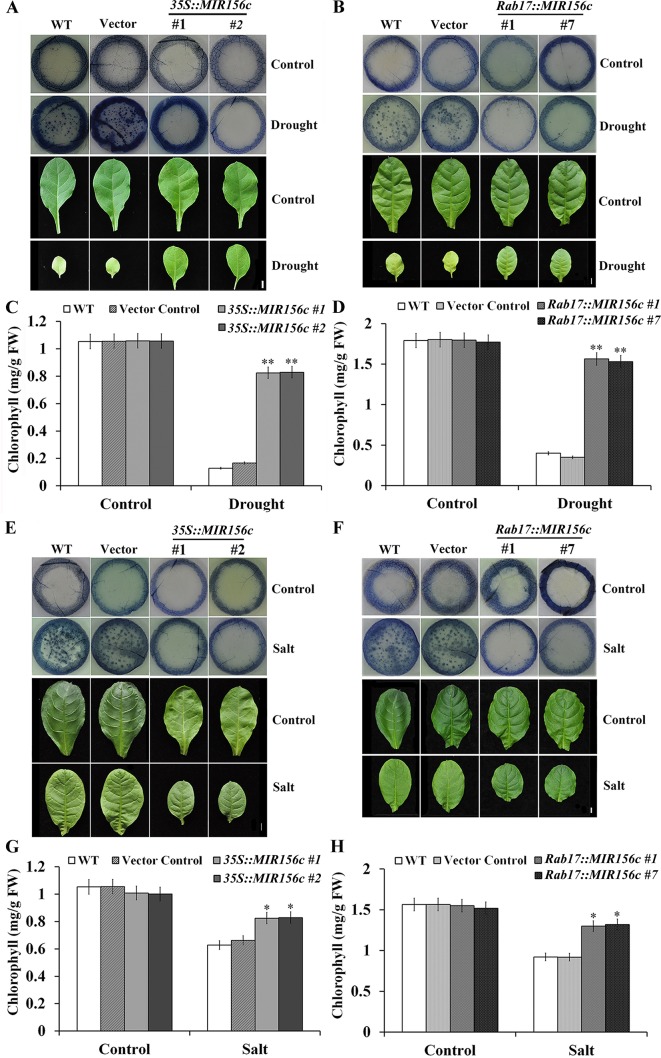
Nitroblue tetrazolium (NBT) staining and chlorophyll content assays of wild type (WT), vector control, and both *35S::MIR156* and *Rab17::MIR156* transgenic plants after drought or salt treatment. **(A)** Leaf disk NBT staining of WT, vector control, and transgenic lines *35S::MIR156#1* and *35S::MIR156#2* plants after the drought treatment. **(B)** Leaf disk NBT staining of WT, vector control, and transgenic lines *Rab17::MIR156#1* and *Rab17::MIR156#7* plants after the drought treatment. **(C**, **D)** Leaf disk chlorophyll content of WT, vector control and transgenic plants in **(A)** and **(B)**, respectively. **(E)** Leaf disk NBT staining of WT, vector control, and transgenic lines *35S::MIR156#1* and *35S::MIR156#2* plants after the salt treatment. **(F)** Leaf disk NBT staining of WT, vector control, and transgenic lines *Rab17::MIR156#1* and *Rab17::MIR156#7* plants after the salt treatment. **(G**, **H)** Leaf disk chlorophyll contents of WT, vector control and transgenic plants in **(E)** and **(F)**, respectively. Data are shown as mean ± SD from three biological replicates. Asterisks indicate significant differences from the corresponding control values at values at *0.01 < P < 0.05 and **P < 0.01. Scale bar = 1 cm.

We further performed nitroblue tetrazolium (NBT; free-radical stain) staining with the leaf disks of wild type, vector control, and *35S::ZmmiR156* and *Rab17::MIR156* transgenic plants grown under both normal and stress conditions. Compared to the leaf disks of wild type and vector control plants, leaf disks of *35S::ZmmiR156* and *Rab17::MIR156* transgenic plants showed a stronger tolerance to the damage caused by oxidative stress (**Figures 5A, B, E, F**). Upon treatment of drought or salt stress, there was significant chlorophyll loss in wild type and vector control plants but only a marginal decrease in *35S::ZmmiR156* and *Rab17::MIR156* transgenic plants (**Figures 5C, D, G, H**). Collectively, these results imply that expression of ZmmiR156 improved resistance to the oxidative stress-induced membrane hydroperoxidation in transgenic tobacco plants.

### Down-Regulated *SPL* and Senescence-Associated Gene Expression in Transgenic Tobacco Plants

**Figure 6:**
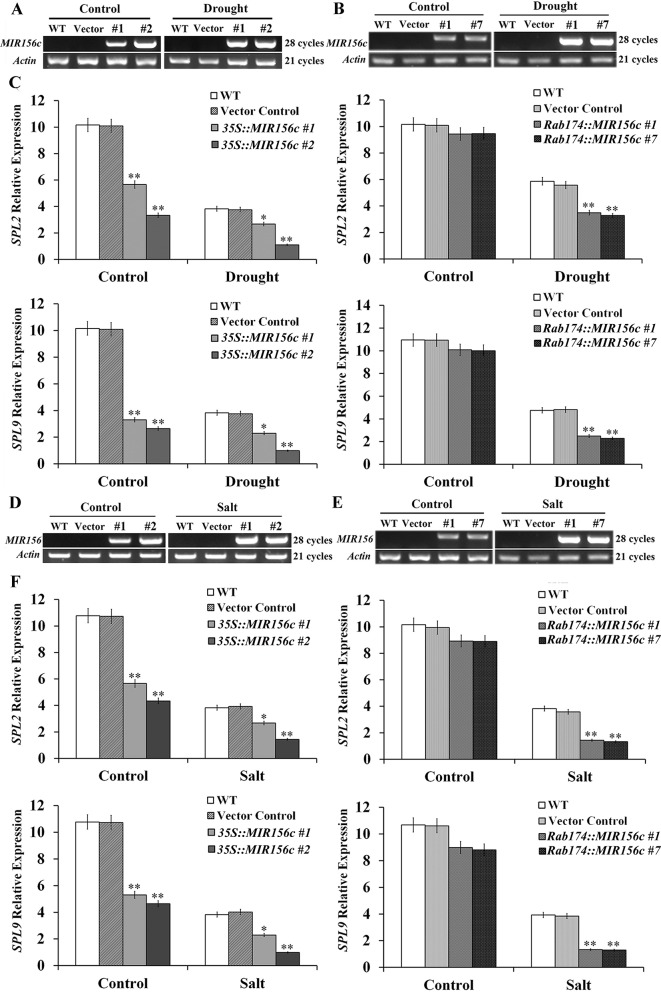
ZmmiR156 and *SPL* gene expression in wild type (WT), vector control, and both *35S::MIR156* and *Rab17::MIR156* transgenic plants after drought or salt treatment. **(A)** RT-PCR analyses of ZmmiR156 expression in WT, vector control, and transgenic lines *35S::MIR156#1* and *35S::MIR156#2* plants grown under normal and drought stress conditions. **(B)** RT-PCR analyses of ZmmiR156 expression in WT, vector control, and transgenic lines *Rab17::MIR156#1* and *Rab17::MIR156#7* plants grown under normal and drought stress conditions. **(C)** Quantitative real-time PCR analyses of *SPL* gene expression in WT, vector control, and transgenic lines *35S::MIR156#1*, *35S::MIR156#2*, *Rab17::MIR156#1*, and *Rab17::MIR156#7* plants grown under normal and drought stress conditions. **(D)** RT-PCR analyses of ZmmiR156 expression in WT, vector control, and transgenic lines *35S::MIR156#1* and *35S::MIR156#2* plants grown under normal and salt stress conditions. **(E)** RT-PCR analyses of ZmmiR156 expression in WT, vector control, and transgenic lines *Rab17::MIR156#1* and *Rab17::MIR156#7* plants grown under normal and salt stress conditions. **(F)** Quantitative real-time PCR analyses of *SPL* gene expression in WT, vector control, and transgenic lines *35S::MIR156#1*, *35S::MIR156#2*, *Rab17::MIR156#1*, and *Rab17::MIR156#7* plants grown under normal and salt stress conditions. The *NtActin* gene was employed as an internal control. Data are shown as mean ± SD from three biological replicates. Asterisks indicate significant differences from the corresponding control values at *P < 0.05, **P < 0.01.

To see if ZmmiR156 expression has been properly manipulated in transgenic tobacco plants, we first investigated the transcript level of ZmmiR156 in wild type, vector control, and *35S::ZmmiR156* and *Rab17::MIR156* transgenic plants. Under either normal or stress condition, no ZmmiR156 expression was detected in wild type and vector control plants, whereas constitutive ZmmiR156 expression was observed in transgenic lines *35S::MIR156#1* and *35S::MIR156#2* (**Figures 6A, D**). Under normal growth condition, although a very low expression of ZmmiR156 was also observed in transgenic lines *Rab17::MIR156#1* and *Rab17::MIR156#7*, the transcription of ZmmiR156 was strongly induced by drought or salt stress (**Figures 6B, E**). We then examined the relative expression levels of *NtSPL2* and *NtSPL9*, the two *SPL* family genes potentially targeted by ZmmiR156 in tobacco. Consistent with the expression of ZmmiR156, *NtSPL2* and *NtSPL9* in transgenic lines *35S::MIR156#1* and *35S::MIR156#2* were drastically down-regulated under both normal and stress conditions, but in transgenic lines *Rab17::MIR156#1* and *Rab17::MIR156#7*, *NtSPL2* and *NtSPL9* were significantly down-regulated only under drought or salt stress conditions (**Figures 6C, F**).

**Figure 7:**
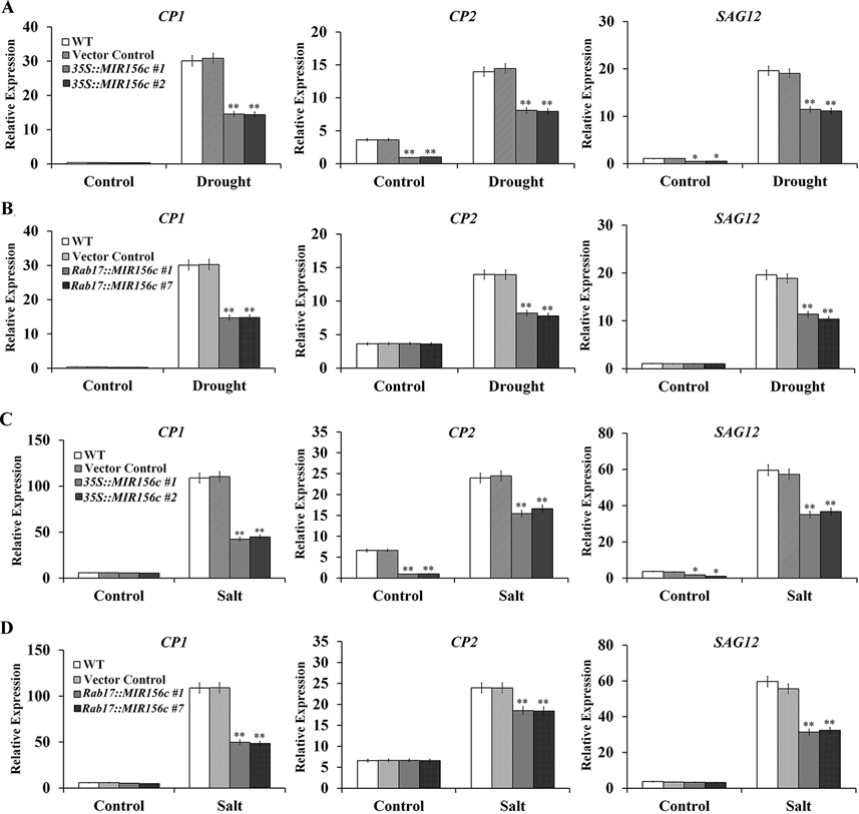
Senescence-associated gene expression in wild type (WT), vector control, and both *35S::MIR156* and *Rab17::MIR156* transgenic plants after drought or salt treatment. **(A)** Quantitative real-time PCR analyses of *CP1*, *CP2*, and *SAG12* expression in WT, vector control and transgenic lines *35S::MIR156#1* and *35S::MIR156#2* plants grown under normal and drought stress conditions. **(B)** Quantitative real-time PCR analyses of *CP1*, *CP2*, and *SAG12* expression in WT, vector control and transgenic lines *Rab17::MIR156#1* and *Rab17::MIR156#7* plants grown under normal and drought stress conditions. **(C)** Quantitative real-time PCR analyses of *CP1*, *CP2*, and *SAG12* expression in WT, vector control and transgenic lines *35S::MIR156#1* and *35S::MIR156#2* plants grown under normal and salt stress conditions. **(D)** Quantitative real-time PCR analyses of *CP1*, *CP2,* and *SAG12* expression in WT, vector control and transgenic lines *Rab17::MIR156#1* and *Rab17::MIR156#7* plants grown under normal and salt stress conditions. The *NtActin* gene was employed as an internal control. Data are shown as mean ± SD from three biological replicates. Asterisks indicate significant differences from the corresponding control values at **P < 0.01.

Senescence is a process of growth and recession at cellular, tissue, organ or whole plant level which is influenced by both internal and external factors (Lim et al., 2007). In this process, the relative expression of a number of genes, especially transcription factors and their downstream target genes involved in metabolism and signal perception, will be up- or down-regulated. To date, senescence-associated genes (*SAGs*) from different plant species have been isolated, including *Arabidopsis*, rice and tobacco (Getu et al., 2006; Lim et al., 2007; Ewa et al., 2009; Branka et al., 2015). We compared the expression of *Cysteine protease 1 (CP1)*, *Cysteine protease 2 (CP2)* and *SAG12* in the leaves of wild type, vector control, and *35S::ZmmiR156* and *Rab17::MIR156* transgenic plants grown under normal and stress conditions. Under normal growth condition, although no significant difference was seen in the expression level of *CP1*, possibly due to its low expression, the expression of *CP2* and *SAG12* was significantly down-regulated in transgenic lines *35S::MIR156#1* and *35S::MIR156#2* (**Figures 7A, C**). No significant difference in expression was observed in transgenic lines *Rab17::MIR156#1* and *Rab17::MIR156#7* (**Figures 7B, D**). Upon treatment with drought or salt stress, expression of *CP1*, *CP2*, and *SAG12* was up-regulated in all the plants, with less significant up-regulation in transgenic lines *35S::MIR156#1*, *35S::MIR156#2*, *Rab17::MIR156#1*, and *Rab17::MIR156#7* (**Figures 7A–D**).

## Discussion

To deal with harmful environmental conditions such as drought and salt stress, plants have developed various systems to incorporate the reprogramming of gene expressions (Zhu, 2002; Chinnusamy et al., 2004). By high-throughput small RNA deep sequencing, a number of drought- and salt-responsive miRNAs involved in these regulatory networks have been identified in different plant species (Hobert, 2008; Yu et al., 2008; Jian et al., 2016; Saminathan et al., 2016). Among them, miR156 has been shown to have an important function in plant response to drought and salinity stresses (Sun et al., 2012; Wang et al., 2013; Bhardwaj et al., 2014).

In addition to its function in plant response to adverse stresses, miR156 also plays a crucial role in plant growth and development. Previously, constitutive expression of miR156 improved the tolerance to abiotic stress, but also affected the regular growth and development of transgenic plants (Wu and Poethig, 2006; Chuck et al., 2007; Schwarz et al., 2008; Wang et al., 2008; Jiao et al., 2010; Wang et al., 2011; Xie et al., 2012; Sun et al., 2019). We aimed to investigate the feasibility of improving resistance to abiotic stress without altering the regular growth and development of transgenic plants by genetically manipulating the expression pattern of miR156. Therefore, two kinds of transgenic tobacco plants with either constitutive or stress-induced expression of ZmmiR156 were generated, and their resistance to drought and salt stress as well as their growth features were compared under both normal and abiotic stress conditions (**Supplementary Figures S3A**–**D**).

Based on the assumption that senescence is a form of cell death programming activated by drought, isopentenyltransferase (IPT) has been expressed in tobacco and successfully improved the tolerance to drought stress by delaying the senescence of transgenic plants (Rivero et al., 2007). Similarly, constitutive expression of miR156 in *Arabidopsis* and rice prolonged the juvenile phase, and improved the resistance of transgenic plants to salt and mannitol stresses (Wu and Poethig, 2006; Wang et al., 2011; Cui et al., 2014). However, detailed analyses on the growth and resistance to abiotic stress at different developmental stages were not carried out. In this work, the relative growth of wild type, vector control, and both *35S::ZmmiR156* and *Rab17::MIR156* transgenic plants at seedling, juvenile and adult stages grown under normal and stress conditions were compared. Consistent with the previously reported transgenic *Arabidopsis* and rice, both *35S::ZmmiR156* and *Rab17::MIR156* transgenic plants showed improved tolerance to salt and mannitol stresses at the early seedling development stages, as indicated by the more vigorous growth of roots and shoots compared to the wild type and relative vector control seedlings (**Figures 1A–F**). Similar results were also observed in transgenic tobacco plants at juvenile and adult stages grown in greenhouse (**Figures 2A–D**, **3A–D**, and **4A–C, F–H**).

As a hydroxyl radical scavenger, proline makes a significant contribution to the adjustment to osmotic stress and the protection of macromolecules during drought and salt stresses (Hong et al., 2000). Upon treatment with drought or salt stress, proline accumulated to a significantly higher level in both *35S::ZmmiR156* and *Rab17::MIR156* transgenic plants than in wild type and vector control plants (**Figures 2E**, **3E**, and **4D, I**). This is consistent with previous reports that transgenic salt tolerant plants accumulated more proline (Nanjo et al., 1999; Zhang and Blumwald, 2001; Zhang et al., 2016). Therefore, the augmented proline accumulation in transgenic plants may have helped protect the activity of antioxidative enzymes and as a result alleviated the adverse impacts imposed by drought and salt on transgenic plants.

One of the major causes of the adverse impacts of drought and salt is the generation of reactive oxygen species (ROS) from chloroplast and mitochondrial metabolism induced by abiotic stress, which causes membrane damage and electrolyte leakage (Apse and Blumwald, 2002). Upon treatment with drought or salt stress, a significant increase of MDA content was observed in wild type and vector control plants (**Figures 2F**, **3F**, and **4E, J**). In addition, a higher chlorophyll content and antioxidant enzyme expression was also observed in both *35S::ZmmiR156* and *Rab17::MIR156* transgenic plants (**Figures 5A–H**, **Supplementary Figures S6A**–**F**, and **7A**–**F**). Therefore, we postulated that under adverse growth conditions, expression of ZmmiR156 helped protect cell membrane integrity in transgenic plants. This hypothesis was also supported by the observations of transgenic tobacco expressing inositol polyphosphate 6-/3-kinase AtIpk2β and heat shock protein LeHSP21 (Yang et al., 2008; Zhang et al., 2016).

At both seedling and juvenile stages, no significant phenotype changes were observed between wild type, vector control, and both *35S::ZmmiR156* and *Rab17::MIR156* transgenic plants under normal growth condition (**Figures 1A–F**, **2A, C, D**, and **3A, C, D**). However, at adult and reproductive stages, transgenic tobacco plants with constitutive expression of ZmmiR156 showed slower growth, delayed flowering and increased branching compared to the wild type, vector control and transgenic tobacco plants with stress-induced expression of ZmmiR156 (**Figures 4A, C, F, G, H**; **Supplementary Figures S4A**, **B**, and **S5A**, **B**). This is consistent with the biological role of ZmmiR156 in plant growth and development. Similar morphological changes were also observed in *Arabidopsis*, rice, maize, soybean, and aspen with increased expression of miR156, and transgenic chickpea constitutively expressing miR408 (Wu and Poethig, 2006; Jiao et al., 2010; Wang et al., 2011; Xie et al., 2012; Cui et al., 2014; Sun et al., 2019).

As a key channel to exchange gas with the outside environment, the stoma regulates the photosynthesis, transpiration and water use of plants (Chaerle et al., 2005). Environmental cues, such as light intensity and quality, water status, temperature and atmospheric carbon dioxide concentration, as well as endogenous signals, control the development, density and aperture of stomata in plants (Hetherington and Woodward, 2003; Saibo et al., 2003). In addition to the altered growth and development, transgenic plants with constitutive expression of ZmmiR156 also showed decreased leaf stomata number (**Supplementary Figures S8A**, **B**). A nearly 66% reduction, leading to a slower water loss, was observed in *35S::MIR156* transgenic plants compared to the wild type, vector control and *Rab17::MIR156* transgenic plants (**Supplementary Figures S8C**, **D**). Further treatment with ABA showed no significant difference in stomatal aperture in all of the plants (**Supplementary Figure S9A**, **B**). We then evaluated the ABA sensitivity of wild type, vector control and *35S::MIR156* transgenic plants during germination and early seedling development. Seeds were sown on MS medium containing different concentrations of ABA. Again, no significant difference was observed after two weeks, as indicated by root lengths and total fresh weights, suggesting that ZmmiR156 may have improved tolerance to abiotic stress in an ABA-independent manner (**Supplementary Figures S10A**–**C**).

Leaf senescence involves the expression of a specific set of SAGs. Since constitutive expression of ZmmiR156 prolonged the juvenile phase of transgenic plants, we compared the expression profiles of *SPL* and *SAG* genes in both *35S::MIR156* and *Rab17::MIR156* transgenic plants. Constitutive expression of ZmmiR156 reduced the expression of miR156-targeted *SPL* and *SAG* genes under both normal and stress conditions, whereas stress-induced expression of ZmmiR156 reduced the expression of miR156-targeted *SPL* and *SAG* genes only under drought or salt stress conditions (**Figures 6C, F**, and **7A–D**). Similar strategy was also used to improve the salt, drought and freezing tolerance of transgenic *Arabidopsis*, wheat and barley plants expressing *DREB* transcription factor genes, driven with the stress inducible *rd29A* or *rab17* promoter (Kasuga et al., 1999; Morran et al., 2011). Taken together, our results demonstrate that ZmmiR156 expression can be temporally engineered to improve plant resistance to abiotic stress without causing any significant effects on the regular growth and development of transgenic plants.

## Data Availability Statement

All datasets generated for this study are included in the article/**Supplementary Material**.

## Author Contributions

TK, C-YY, YL, X-TG, BL, W-MS, and YB performed the experiments and analyzed the data. TK, H-XZ, and BL conceived the study. TK, C-YY, H-XZ, and BL wrote the manuscript. All authors read and agreed at the last version of the manuscript.

## Funding

This work was supported by the following grants: the National Key R & D Program of China (2016YFD0600106); The Modern Agricultural Industry Technology System Innovation Team of Shandong Province of China (SDAIT-02-05); The Shandong Agricultural Elite Variety Project (2019LZGC010); The Natural Science Foundation of Shandong Province of China (ZR2016CB48); The National Key Program on Transgenic Research (2018ZX08020002-003-004); The National Natural Science Foundation of China (31700524, 31870576); and The Science and Technology Develop Project in Yantai (2018XSCC041).

## Conflict of Interest

The authors declare that the research was conducted in the absence of any commercial or financial relationships that could be construed as a potential conflict of interest.

## References

[B1] Apse M. P. Blumwald E. (2002). Engineering salt tolerance in plants. Curr. Opin. Biotechnol. 13, 146–150. 10.1016/S0958-1669(02)00298-7 11950567

[B2] Arshad M. Gruber M. Y. Wall K. Hannoufa A. (2017). An insight into microRNA156 Role in salinity stress responses of alfalfa. Front. Plant Sci. 8, 356. 10.3389/fpls.2017.00356 28352280 PMC5348497

[B3] Axtell M. J. Bowman J. L. (2008). Evolution of plant microRNAs and their targets. Trends Plant Sci. 13, 343–349. 10.1016/j.tplants.2008.03.009 18502167

[B4] Bartel D. P. (2004). MicroRNAs: Genomics, biogenesis, mechanism, and function. Cell 116, 281–297. 10.1016/S0092-8674(04)00045-5 14744438

[B5] Bates L. S. Walden R. P. (1973). Rapid determination of free proline for water stress studies. Plant Soil 39, 205–207. 10.1007/BF00018060

[B6] Baulcombe D. (2004). RNA silencing in plants. Nature 431, 356–363. 10.1038/nature02874 15372043

[B7] Bhardwaj A. R. Joshi G. Pandey R. Kukreja B. Goel S. Jagannath A. (2014). A genome-wide perspective of miRNA in response to high temperature, salinity and drought stresses in *brassica juncea* (Czern) L. PloS One 9, e92456. 10.1371/journal.pone.0092456 24671003 PMC3966790

[B8] Branka U. Dušica J. Ana S. Václav M. Petre I. D. Snežana B. (2015). Characterization of natural leaf senescence in tobacco (*Nicotiana tabacum*) plants grown *in vitro* . Protoplasma 253, 259–275. 10.1007/s00709-015-0802-9 25837009

[B9] Carrington J. C. Ambros V. (2003). Role of MicroRNAs in plant and animal development. Science 301, 336–338. 10.1126/science.1085242 12869753

[B10] Chaerle L. Saibo N. Straeten D. V. D. (2005). Tuning the pores: towards engineering plants for improved water use efficiency. Trends Biotechnol. 23, 308–315. 10.1016/j.tibtech.2005.04.005 15922083

[B11] Chinnusamy V. Schumaker K. Zhu J. K. (2004). Molecular genetic perspectives on cross-talk and specificity in abiotic stress signaling in plants. J. Exp. Bot. 55, 225–236. 10.1093/jxb/erh005 14673035

[B12] Chuck G. Cigan A. M. Saeteurn K. Hake S. (2007). The heterochronic maize mutant *Corngrass1* results from overexpression of a tandem microRNA. Nat. Genet. 39, 544–549. 10.1038/ng2001 17369828

[B13] Cui L. G. Shan J. X. Shi M. Gao J. P. Lin H. X. (2014). The *miR156-SPL9-DFR* pathway coordinates the relationship between development and abiotic stress tolerance in plants. Plant J. 80, 1108–1117. 10.1111/tpj.12712 25345491

[B14] Ewa N. Lisa P. Christine D. Piotr R. Zbigniew M. Karin K. (2009). Spatial patterns of senescence and development-dependent distribution of reactive oxygen species in tobacco (*Nicotiana tabacum*) leaves. J. Plant Physiol. 166, 1057–1068. 10.1016/j.jplph.2008.12.014 19261356

[B15] Fryer M. J. Oxborough K. Mullineaux P. M. Baker N. R. (2002). Imaging of photo-oxidative stress responses in leaves. J. Exp. Bot. 53, 1249–1254. 10.1093/jxb/53.3721249 11997373

[B16] Gandikota M. Birkenbihl R. P. Höhmann S. Cardon G. H. Saedler H. Huijser P. (2007). The miRNA156/157 recognition element in the 3′ UTR of the *Arabidopsis* SBP box gene SPL3 prevents early flowering by translational inhibition in seedlings. Plant J. 49, 683–693. 10.1111/j.1365-313X.2006.02983.x 17217458

[B17] Getu B. Christine H. F. Karl J. K. (2006). Two new cysteine proteinases with specific expression patterns in mature and senescent tobacco (*Nicotiana tabacum* L.) leaves. J. Exp. Bot. 57, 1431–1443. 10.1093/jxb/erj123 16551685

[B18] Hajyzadeh M. Turktas M. Khawar K. M. Unver T. (2015). miR408 overexpression causes increased drought tolerance in chickpea. Gene 555, 186–193. 10.1016/j.gene.2014.11.002 25445265

[B19] He L. Hannon G. J. (2004). MicroRNAs: small RNAs with a big role in gene regulation. Nat. Rev. Genet. 5, 522–531. 10.1038/nrg1379 15211354

[B20] Hetherington A. M. Woodward F. I. (2003). The role of stomata in sensing and driving environmental change. Nature 424, 901–908. 10.1038/nature01843 12931178

[B21] Hobert O. (2008). Gene regulation by transcription factors and MicroRNAs. Science 319, 1785–1786. 10.1126/science.1151651 18369135

[B22] Hong Z. L. Lakkineni K. Zhang Z. M. Verma D. P. S. (2000). Removal of feedback inhibition of DELTA1-pyrroline-5-carboxylate synthetase results in increased proline accumulation and protection of plants from osmotic stress. Plant Physiol. 122, 1129–1136. 10.1104/pp.122.41129 10759508 PMC58947

[B23] Jian H. J. Wang J. Wang T. Y. Wei L. J. Li J. N. Liu L. Z. (2016). Identification of rapeseed microRNAs involved in early stage seed germination under salt and drought stresses. Front. Plant Sci. 7, 658. 10.3389/fpls.2016.00658 27242859 PMC4865509

[B24] Jiao Y. Q. Wang Y. H. Xue D. W. Wang J. Yan M. X. Liu G. F. (2010). Regulation of *OsSPL14* by OsmiR156 defines ideal plant architecture in rice. Nat. Genet. 42, 541–544. 10.1038/ng.591 20495565

[B25] Kantar M. Unver T. Budak H. (2010). Regulation of barley miRNAs upon dehydration stress correlated with target gene expression. Funct. Integr. Genom. 10, 493–507. 10.1007/s10142-010-0181-4 20676715

[B26] Kasuga M. Liu Q. Miura S. Yamaguchi-Shinozaki K. Shinozaki K. (1999). Improving plant drought, salt, and freezing tolerance by gene transfer of a single stress-inducible transcription factor. Nat. Biotechnol. 17 (3), 287–291. 10.1038/7036 10096298

[B27] Li B. Liu H. Zhang Y. Kang T. Zhang L. Tong J. H. (2013). Constitutive expression of cell-wall invertase genes increase grain yield and starch content in maize. Plant Biotechnol. J. 11, 1080–1091. 10.1111/pbi.12102 23926950

[B28] Li W. Wang T. Zhang Y. Li Y. (2016). Overexpression of soybean miR172c confers tolerance to water deficit and salt stress, but increases ABA sensitivity in transgenic *Arabidopsis thaliana* . J. Exp. Bot. 67, 175–194. 10.1093/jxb/erv450 26466661

[B29] Lichtenthaler H. K. (1987). Chlorophylls and carotenoids: pigments of photosynthetic biomembranes. Methods Enzymol. 148, 350–382. 10.1016/0076-6879(87)48036-1

[B30] Lim P. O. Kim H. J. Nam H. G. (2007). Leaf senescence. Annu. Rev. Plant Biol. 58, 115–136. 10.1146/annurev.arplant.57.032905.105316 17177638

[B31] Martin R. C. Asahina M. Liu P. P. Kristof J. R. Coppersmith J. L. Pluskota W. E. (2010). The microRNA156 and microRNA172 gene regulation cascades at post-germinative stages in *Arabidopsis* . Seed Sci. Res. 20, 79–87. 10.1017/S0960258510000085

[B32] Morran S. Eini O. Pyvovarenko T. Parent B. Singh R. Ismagul A. (2011). Improvement of stress tolerance of wheat and barley by modulation of expression of DREB/CBF factors. Plant Biotechnol. J. 9, 230–249. 10.1111/j.1467-7652.2010.00547.x 20642740

[B33] Murashige T. Skoog F. (1962). A revised medium for rapid growth and bioassays with tobacco tissue cultures. Physiol. Plant 15, 473–495. 10.1111/j.1399-3054.1962.tb08052.x

[B34] Nanjo T. Kobayashi M. Yoshiba Y. Kakubari Y. Yamaguchi-Shinozaki K. Shinozaki K. (1999). Antisense suppression of proline degradation improves tolerance to freezing and salinity in *Arabidopsis thaliana* . FEBS Lett. 461, 205–210. 10.1016/S0014-5793(99)01451-9 10567698

[B35] Rivero R. M. Kojima M. Gepstein A. Sakakibara H. Mittler R. Gepstein S. (2007). Delayed leaf senescence induces extreme drought tolerance in a flowering plant. Proc. Natl. Acad. Sci. U.S.A. 104, 19631–19636. 10.1073/pnas.0709453104 18048328 PMC2148340

[B36] Saibo N. J. Vriezen W. H. Beemster G. T. S. Straeten D. V. D. (2003). Growth and stomata development of *Arabidopsis* hypocotyls are controlled by gibberellins and modulated by ethylene and auxins. Plant J. 33, 989–1000. 10.1046/j.1365-313X.2003.01684.x 12631324

[B37] Saminathan T. Bodunrin A. Singh N. V. Devarajan R. Nimmakayala P. Jeff M. (2016). Genome-wide identification of microRNAs in pomegranate (*Punica granatum L.*) by high-throughput sequencing. BMC Plant Biol. 16, 122. 10.1186/s12870-016-0807-3 27230657 PMC4880961

[B38] Schobert B. (1997). Is there an osmotic regulatory mechanism in algae and higher plants? J. Theor. Biol. 68, 17–26. 10.1016/0022-5193(77)90224-7 916702

[B39] Schwarz S. Grande A. V. Bujdoso N. Saedler H. Huijser P. (2008). The microRNA regulated SBP-box genes *SPL9* and *SPL15* control shoot maturation in *Arabidopsis* . Plant Mol. Biol. 67, 183–195. 10.1007/s11103-008-9310-z 18278578 PMC2295252

[B40] Stief A. Altmann S. Hoffmann K. Pant B. D. Scheible W. R. Baurle I. (2014). *Arabidopsis* miR156 regulates tolerance to recurring environmental stress through *SPL* transcription factors. Plant Cell 26, 1792–1807. 10.1105/tpc.114.123851 24769482 PMC4036586

[B41] Sun G. Stewart C. N. Jr. Xiao P. Zhang B. H. (2012). MicroRNA expression analysis in the cellulosic biofuel crop switchgrass (*Panicum virgatum*) under abiotic stress. PloS One 7, e32017. 10.1371/journal.pone.0032017 22470418 PMC3314629

[B42] Sun Z. Su C. Yun J. Jiang Q. Wang L. Wang Y. (2019). Genetic improvement of the shoot architecture and yield in soya bean plants *via the* manipulation of *GmmiR156b* . Plant Biotechnol. J. 17, 50–62. 10.1111/pbi.12946 29729214 PMC6330639

[B43] Wang H. Wang H. Y. (2015). The miR156/SPL module, a regulatory hub and versatile toolbox, gears up crops for enhanced agronomic traits. Mol. Plant 8, 677–688. 10.1016/j.molp.2015.01.008 25617719

[B44] Wang J. W. Schwab R. Czech B. Mica E. Weigel D. (2008). Dual effects of miR156-targeted *SPL* genes and *CYP78A5/KLUH* on plastochron length and organ size in *Arabidopsis thaliana* . Plant Cell 20, 1231–1243. 10.1105/tpc.108.058180 18492871 PMC2438454

[B45] Wang J. W. Park M. Y. Wang L. J. Koo Y. Chen X. Y. Weigel D. (2011). MiRNA control of vegetativephase change in trees. PloS Genet. 7, e1002012. 10.1371/journal.pgen.1002012 21383862 PMC3044678

[B46] Wang M. Wang Q. L. Zhang B. L. (2013). Response of miRNAs and their targets to salt and drought stresses in cotton (*Gossypium hirsutum* L.). Gene 530, 26–32. 10.1016/j.gene.2013.08.009 23948080

[B47] Wang Y. L. Wu F. J. Bai J. J. He Y. K. (2014). *BrpSPL9* (*Brassicarapa* ssp *pekinensis SPL9*) controls the earliness of heading time in Chinese cabbage. Plant Biotechnol. J. 12, 312–321. 10.1111/pbi.12138 24237584

[B48] Wu G. Poethig R. S. (2006). Temporal regulation of shoot development in *Arabidopsis thaliana* by *Mir156* and its target *SPL3* . Development 133, 3539–3547. 10.1242/dev.02521 16914499 PMC1610107

[B49] Xie K. Shen J. Hou X. Yao J. Li X. Xiao J. (2012). Gradual increase of miR156 regulates temporal expression changes of numerous genes during leaf development in rice. Plant Physiol. 158, 1382–1394. 10.1104/pp.111.190488 22271747 PMC3291253

[B50] Xiong L. Schumaker K. S. Zhu J. K. (2002). Functional and phylogenetic analysis of a DREB/CBF-like gene in perennial ryegrass (*Lolium perenne* L.). Planta 224, 878–888. 10.1007/s00425-006-0273-5 16614820

[B51] Yamaguchi-Shinozaki K. Shinozaki K. (2006). Transcriptional regulatory networks in cellular responses and tolerance to dehydration and cold stresses. Annu. Rev. Plant Biol. 57, 781–803. 10.1146/annurev.arplant.57.032905.105444 16669782

[B52] Yang L. Tang R. J. Zhu J. Q. Liu H. Mueller-Roeber B. Xia H. J. (2008). Enhancement of stress tolerance in transgenic tobacco plants constitutively expressing *AtIpk2β*, an inositol polyphosphate 6-/3-kinase from *Arabidopsis thaliana* . Plant Mol. Biol. 66, 329–343. 10.1007/s11103-007-9267-3 18165921 PMC2238787

[B53] Yoshimura K. Miyao K. Gaber A. Takeda T. Kanaboshi H. Miyasaka H. (2004). Enhancement of stress tolerance in transgenic tobacco plants overexpressing Chlamydomonas glutathione peroxidase in chloroplasts or cytosol. Plant J. 37, 21–33. 10.1046/j.1365-313X.2003.01930.x 14675429

[B54] Yu X. P. Lin J. Zack D. J. Mendell J. T. Qian J. (2008). Analysis of regulatory network topology reveals functionally distinct classes of microRNAs. Nucleic Acids Res. 36, 6494–6503. 10.1093/nar/gkn712 18927108 PMC2582613

[B55] Zhang H. X. Blumwald E. (2001). Transgenic salt-tolerant tomato plants accumulate salt in foliage but not in fruit. Nat. Biotechnol. 19, 765–768. 10.1038/90824 11479571

[B56] Zhang Z. Lin H. Shen Y. Gao J. Xiang K. Liu L. (2012). Cloning and characterization of miRNAs from maize seedling roots under low phosphorus stress. Mol. Biol. Rep. 39, 8137–8146. 10.1007/s11033-012-1661-5 22562381 PMC3383953

[B57] Zhang J. Chen H. Y. Wang H. H. Li B. Yi Y. J. Kong F. J. (2016). Constitutive expression of a tomato small heat shock protein gene *LeHSP21* improves tolerance to high temperature stress by enhancing antioxidation capacity in tobacco. Plant Mol. Biol. Rep. 34, 399–409. 10.1007/s11105-015-0925-3

[B58] Zhu J. K. (2002). Salt and drought stress signal transduction in plants. Annu. Rev. Plant Biol. 53, 247–273. 10.1146/annurev.arplant.53.091401.143329 12221975 PMC3128348

