# Supplementary material for: Subtly Manipulated Expression of ZmmiR156 in Tobacco Improves Drought and Salt Tolerance Without Changing the Architecture of Transgenic Plants
Source: Front Plant Sci. 2020 Jan 10;10:1664. doi: 10.3389/fpls.2019.01664 (PMC6965348; doi:10.3389/fpls.2019.01664)
Supplement: Supplementary file 1 [file Presentation_1.pdf]

SUPPLEMENTARY MATERIALS

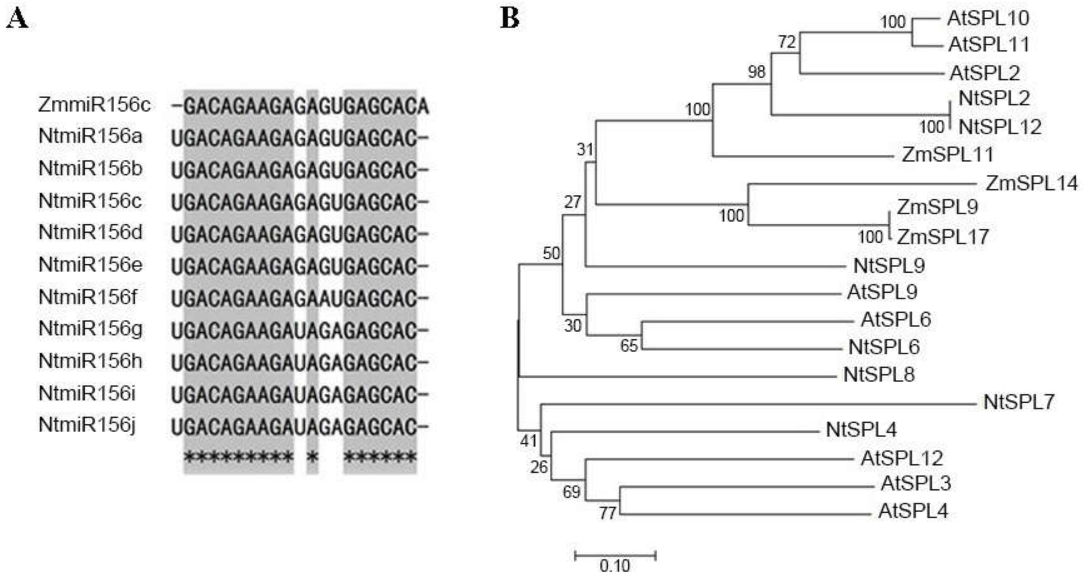

Supplementary Figure S1. Sequence alignment of mature miR156s and phylogenetic analysis of *SPL*s from different plant species. (A) Alignment analyses of mature NtmiR156 and ZmmiR156(c) in maize and tobacco. The same sequences were highlighted in gray color and indicated with asterisks. (B) Phylogenetic analysis of *AtSPLs*, *NtSPLs* and *ZmSPLs*. At, *Arabidopsis thaliana*; Nt, *Nicotiana tabacum*; Zm, *Zea mays*.

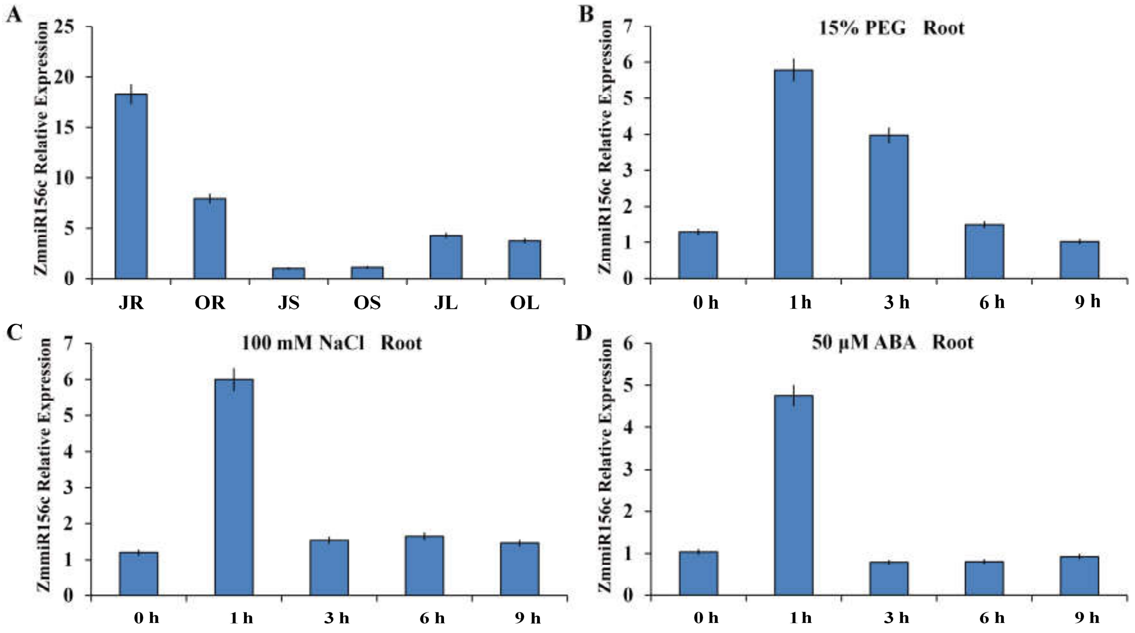

Supplementary Figure S2. ZmmiR156 expression analyses. (A) ZmmiR156 expression in different tissues of juvenile (one-month-old maize) and old (four-month-old) maize plants. (B-D) ZmmiR156 expression in the roots of two-week-old seedlings after treated with 15% PEG, 100 mM NaCl or 50 uM ABA for 0, 1, 3, 6 and 9 hours, respectively. The *ZmActin* gene was employed as an internal control. Data are shown as mean ± SD from three biological replicates. JR, roots from juvenile plants; OR, roots from old plants; JS, stems from juvenile plants; OS, stems from old plants; JL, Leaves from juvenile plants; OL, leaves from old plants.

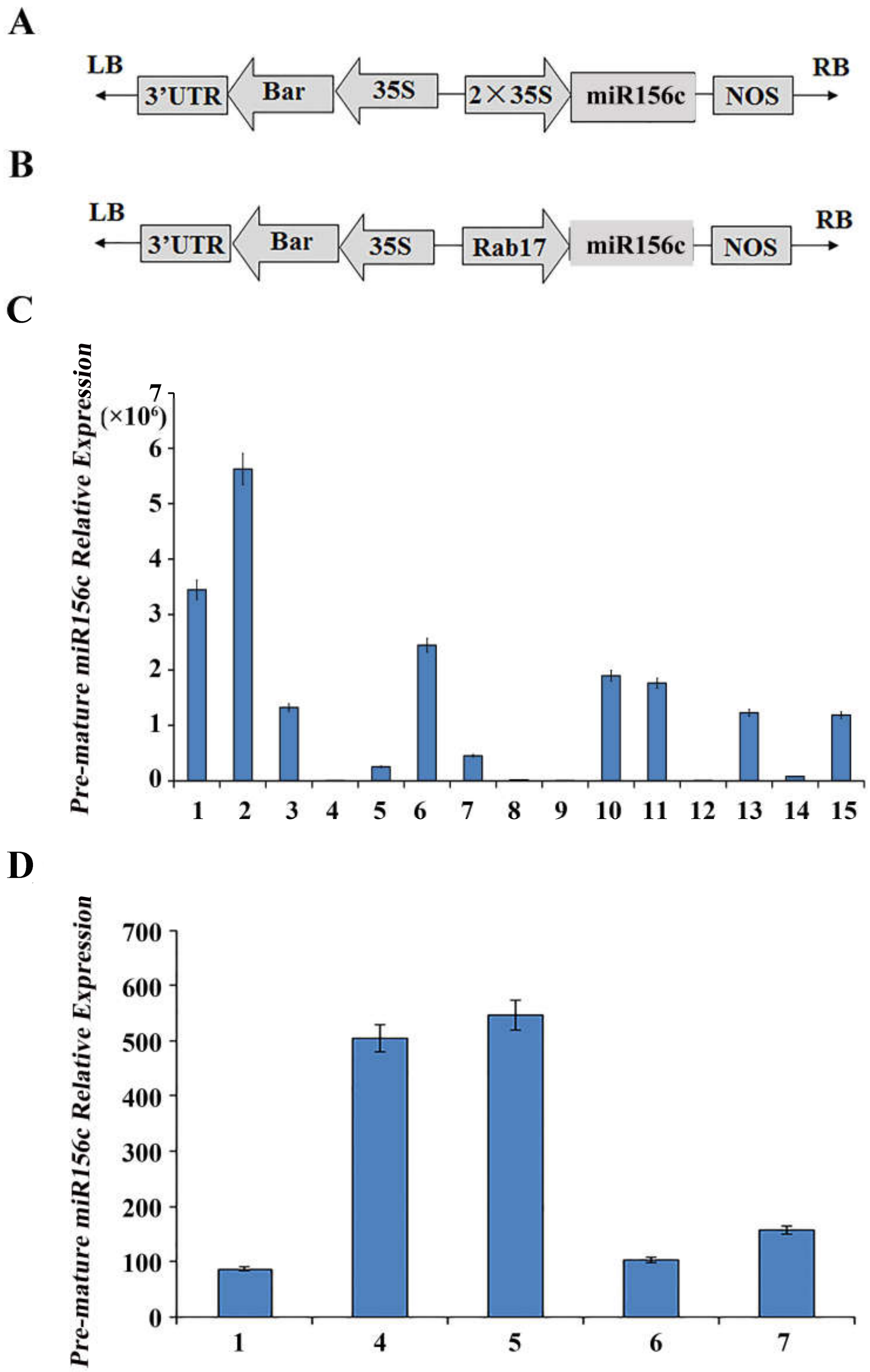

Supplementary Figure S3. Plant expression vectors and molecular confirmation of ZmmiR156 expression in transgenic tobacco plants. (A, B) Schematic maps

of the 35S::MIR156 and Rab17::MIR156 vectors used for tobacco transformation. Expression of ZmmiR156 is driven by two copies of the cauliflower mosaic virus 35S promoter or the drought- and ABA-inducible Rab17 promoter. 35S, CaMV35S promoter; NOS, NOS terminator; Rab17, ZmRab17 promoter. (C) Quantitative real-time PCR analyses of premature ZmmiR156 levels in different *35S::MIR156* transgenic lines. (D) Quantitative real-time PCR analyses of premature ZmmiR156 levels in different *Rab17::MIR156* transgenic lines. Total RNAs were isolated from four-week-old tobacco plants. The *NtActin* gene was employed as an internal control. Data are shown as mean ± SD from three biological replicates.

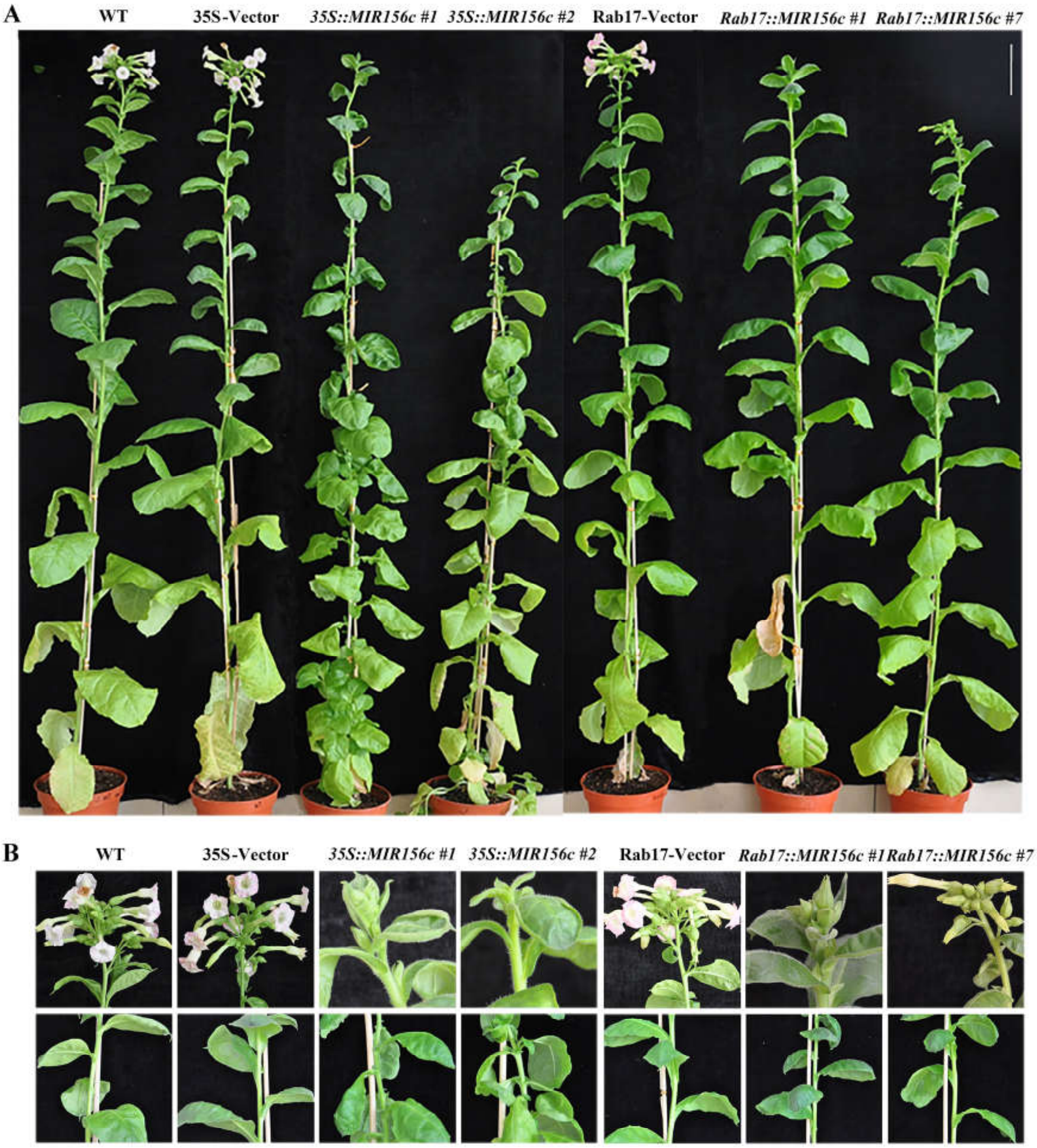

Supplementary Figure S4. Phenotype comparison of wild type (WT), vector control, and *35S::MIR156* and *Rab17::MIR156* transgenic plants grown under normal condition. (A) Phenotypes of six-month-old plants grown in greenhouse. Scale bar = 10 cm. (B) Close-up views to show the delayed flowering and promoted branching in transgenic lines *35S::MIR156#1* and *35S::MIR156#2*.

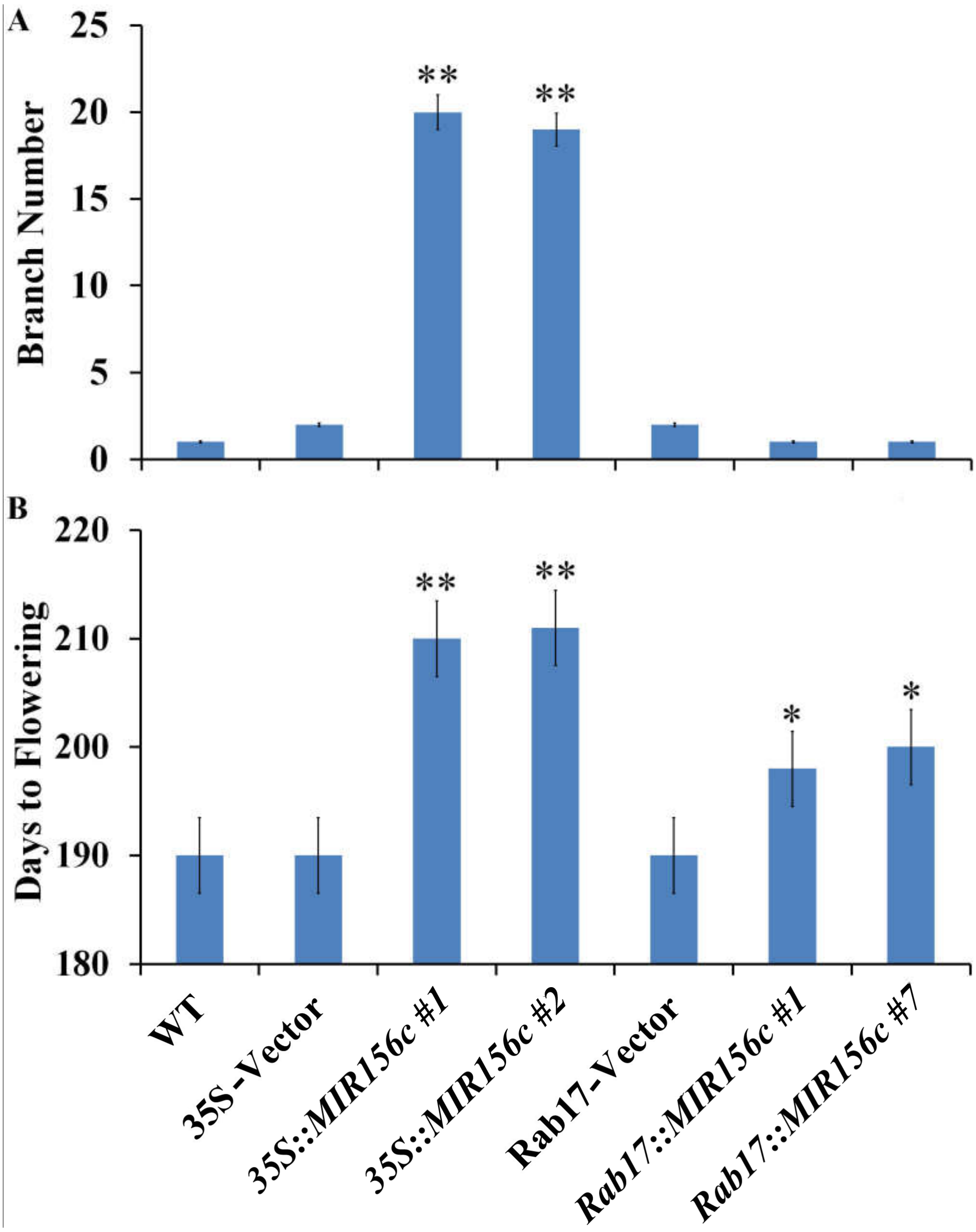

Supplementary Figure S5. Branch numbers and days to flowering of six-month-old wild type (WT), vector control, and both *35S::MIR156* and *Rab17::MIR156* transgenic plants grown under normal condition. (A) Branch numbers. (B) Days to flowering. Data are shown as mean ± SD from three biological replicates. Asterisks indicate significant differences from the corresponding control values at $*0.01 < P < 0.05$ and $**P < 0.01$.

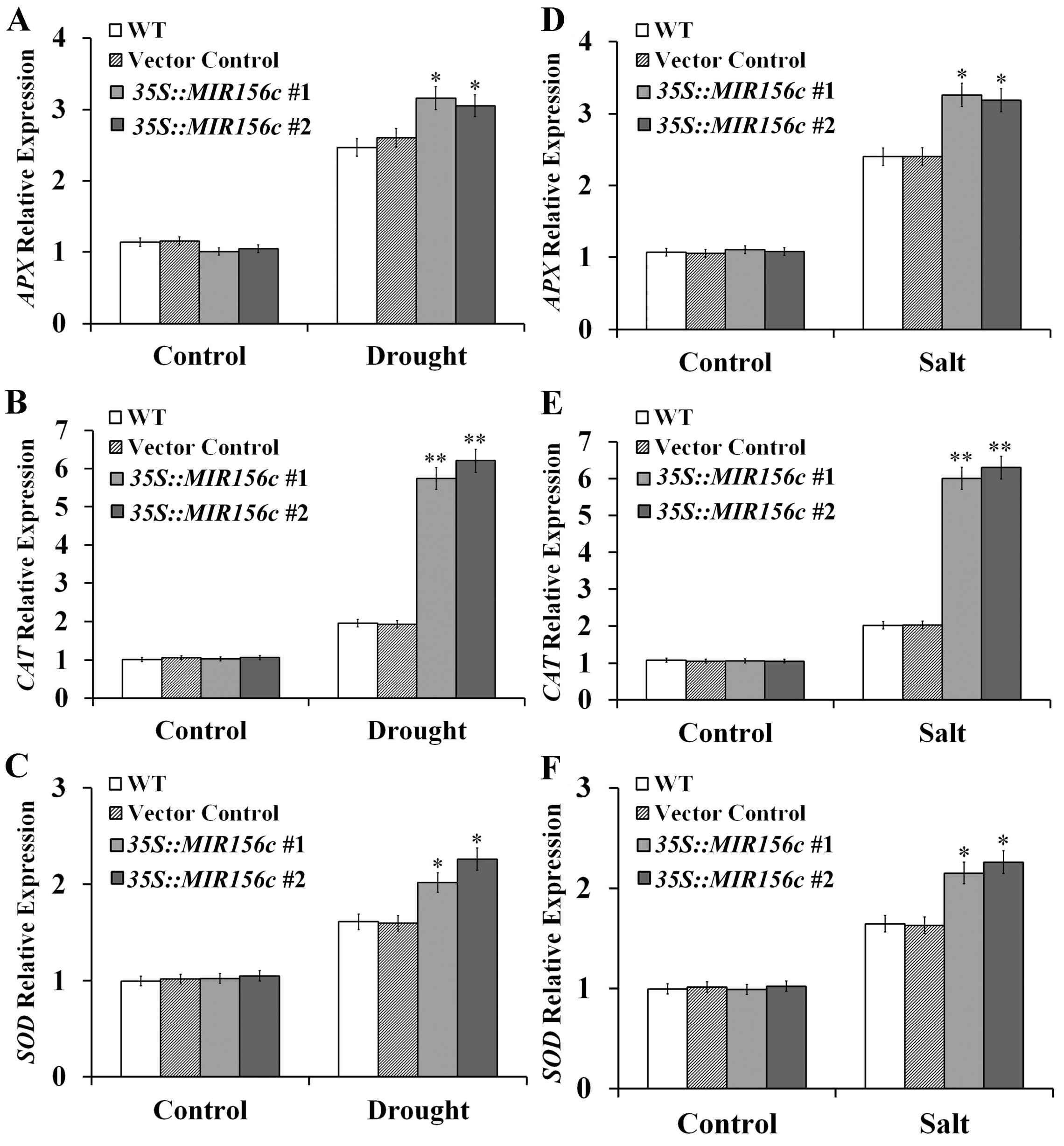

Supplementary Figure S6. Expression of antioxidant genes in wild type (WT), vector control and *35S::MIR156* transgenic plants after treated with drought or salt stress. (A-C) Quantitative real-time PCR analyses of *APX*, *CAT* and *SOD* expression in WT, vector control, and transgenic lines *35S::MIR156#1* and *35S::MIR156#2* plants grown under normal and drought stress conditions. (D-F) Quantitative real-time PCR analyses of

APX, *CAT* and *SOD* expression in WT, vector control, and transgenic lines *35S::MIR156#1* and *35S::MIR156#2* plants grown under normal and salt stress conditions. The *NtActin* gene was employed as an internal control. Data are shown as mean ± SD from three biological replicates. Asterisks indicate significant differences from the corresponding control values at *P < 0.05, **P < 0.01.

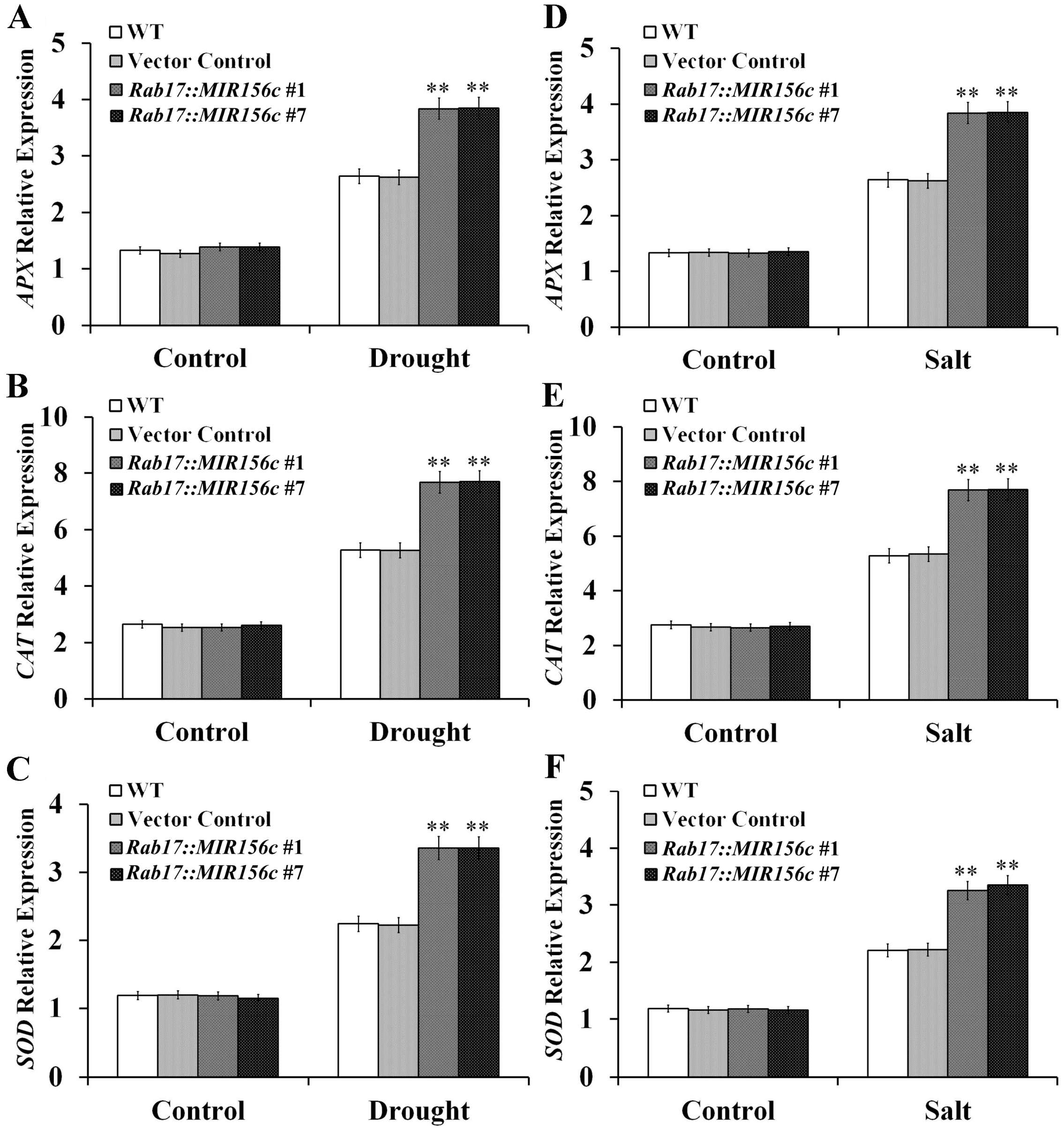

Supplementary Figure S7. Expression of antioxidant genes in wild type (WT), vector control and *Rab17::MIR156* transgenic plants after treated with drought or salt stress. (A-C) Quantitative real-time PCR analyses of *APX*, *CAT* and *SOD* expression in WT, vector control, and transgenic lines *Rab17::MIR156#1* and *Rab17::MIR156#7* plants grown under normal and drought stress conditions. (D-F) Quantitative real-time PCR analyses of *APX*, *CAT* and *SOD* expression in WT, vector control, and transgenic lines

Rab17::MIR156#1 and *Rab17::MIR156#7* plants grown under normal and salt stress conditions. The *NtActin* gene was employed as an internal control. Data are shown as mean ± SD from three biological replicates. Asterisks indicate significant differences from the corresponding control values at $^{**}P < 0.01$.

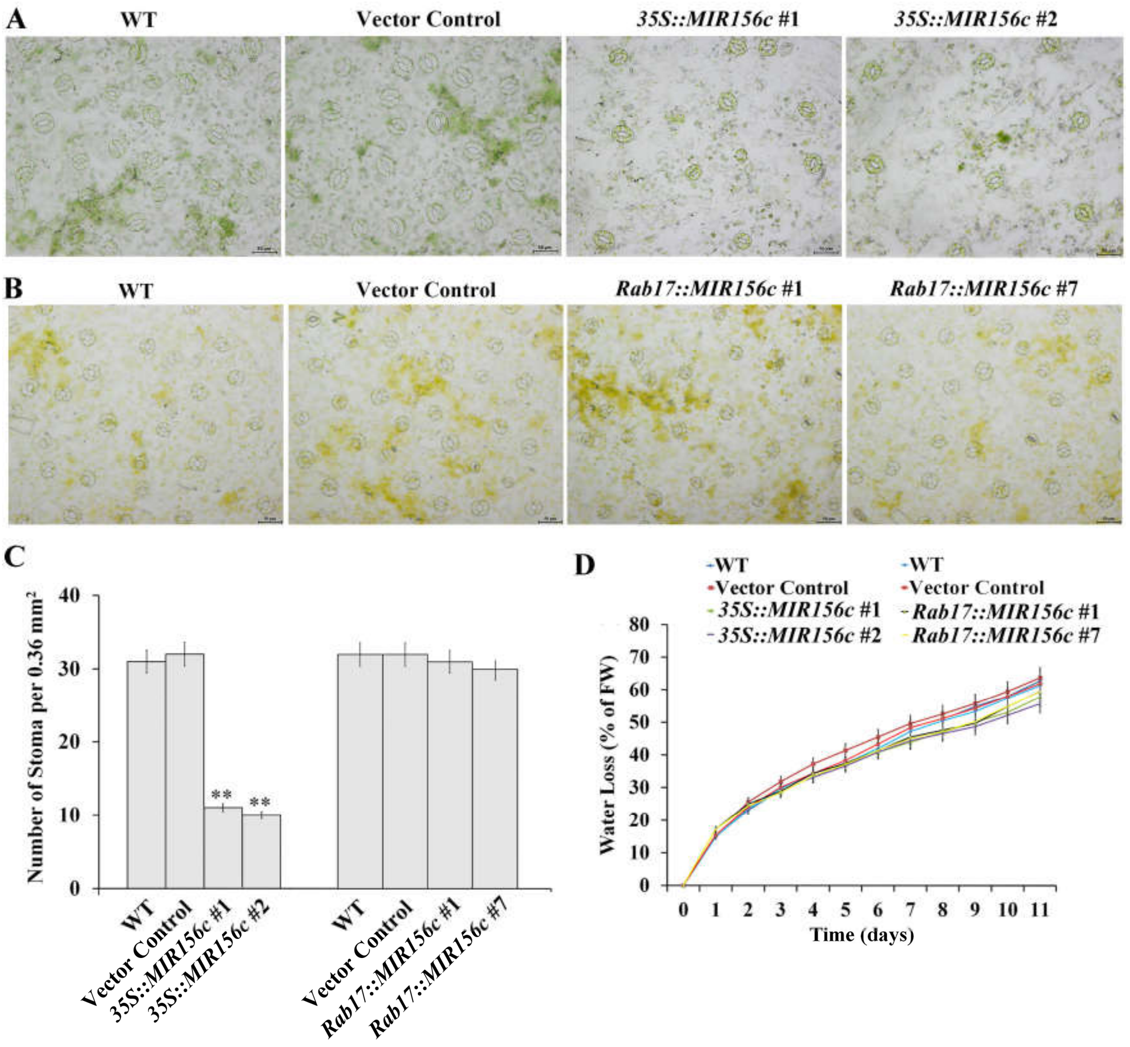

Supplementary Figure S8. Stomatal density and water loss assays. Epidermis peeled from two-month-old plants of wild type (WT), vector control, and both *35S::MIR156* and *Rab17::MIR156* transgenic plants were soaked in stomata-opening solution. (A) Stoma observations of wild type, vector control, and transgenic lines *35S::MIR156#1* and *35S::MIR156#2* plants. Scale bar = 50 µm. (B) Stoma observations of wild type, vector control, and transgenic lines *Rab17::MIR156#1* and *Rab17::MIR156#7* plants. Scale bar = 50 µm. (C) Stomatal density assays. The number of stoma per

0.36 mm^2 of epidermal peels was counted. (D) Leaf water loss tests. Data are shown as mean ± SD from three biological replicates. Asterisks indicate significant differences from the corresponding control values at **$P < 0.01$.

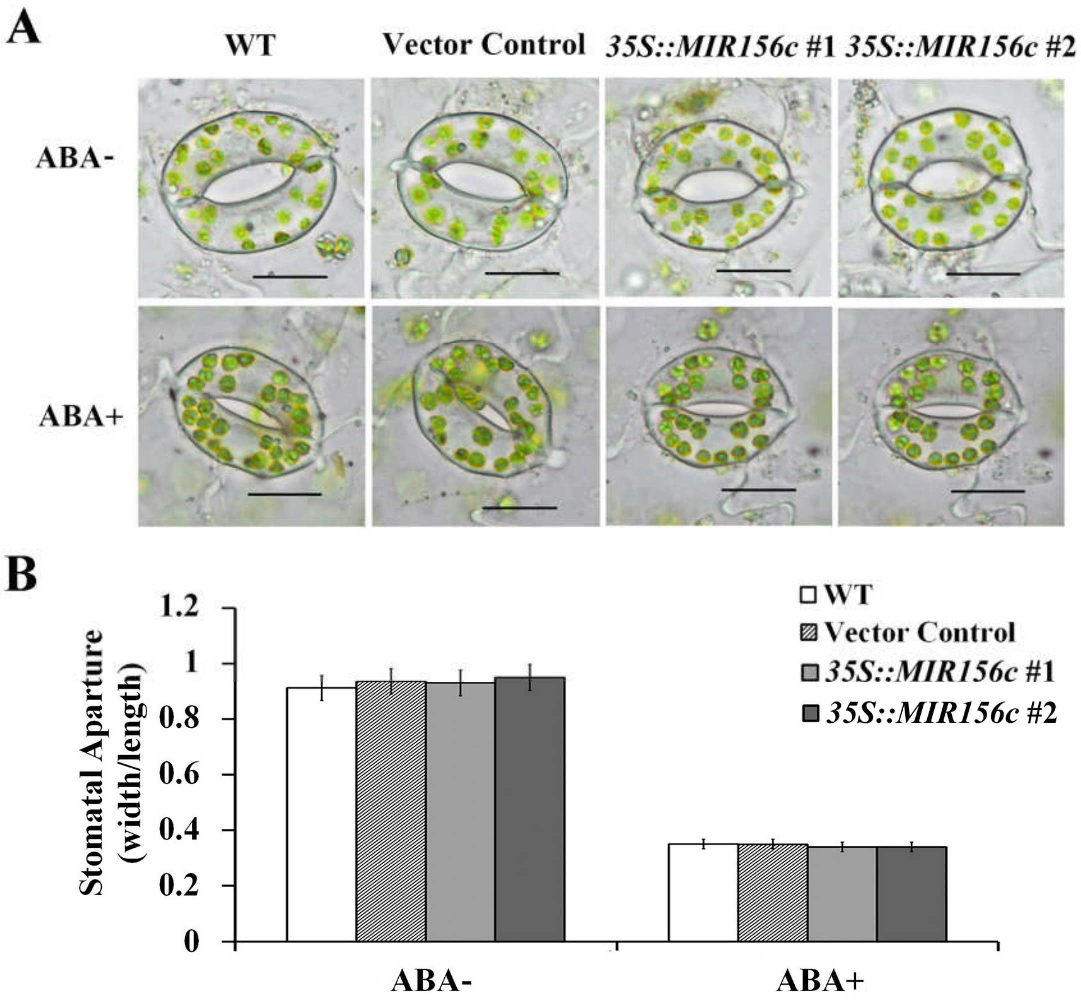

Supplementary Figure S9. Stomatal aperture analyses upon ABA treatment. (A) Comparison of ABA-induced stomatal apertures of wild type, vector control, and transgenic lines *35S::MIR156#1* and *35S::MIR156#2*. Epidermis peeled from two-month-old plants of different genotypes were incubated under light in stomata-opening solution for 3 h and then treated with 0 or 50 μM ABA for 2 hours. Scale bar = 10 μm. (B) Measurements of stomatal aperture in response to ABA. Data are shown as mean ± SD from three biological replicates.

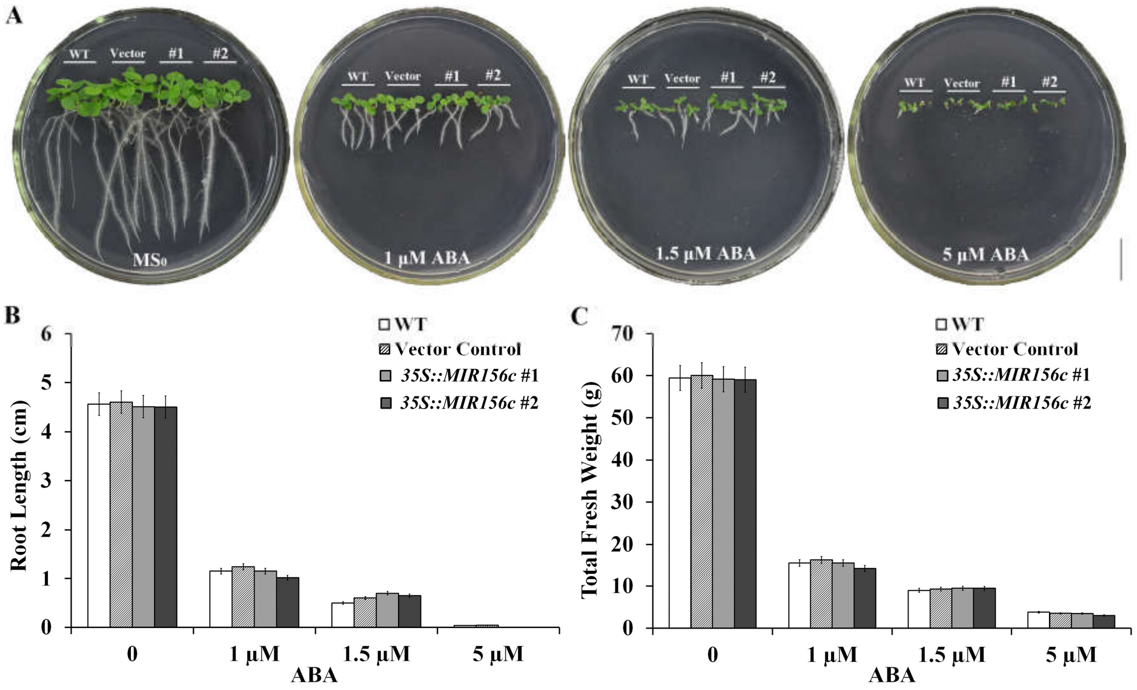

Supplementary Figure S10. Germination analyses of wild type (WT), vector control, and transgenic lines *35S::MIR156#1* and *35S::MIR156#2* plants upon ABA treatment. (A) Seeds were germinated on MS medium containing different concentrations of ABA and cultured vertically for two weeks. Scale bar = 1 cm. (B, C) Root lengths and fresh weights of two-week-old seedlings. Data are shown as mean ± SD from three biological replicates.

Supplementary Table S1. GenBank accession or gene model numbers.

Gene name	GenBank accession number/gene models
ZmActin	AJ01238.1
NtActin	EU938079.1
ZmmiR156	EF541486.1
AtSPL2	NM_123693
AtSPL3	NM_128940
AtSPL4	NM_104194
AtSPL6	AJ011643
AtSPL9	NM_129782
AtSPL10	NM_001036019
AtSPL11	NM_001084134
AtSPL12	NM_115866
NtSPL2	KP260634
NtSPL4	KP260635
NtSPL6	NM_001325188
NtSPL7	NM_001326256
NtSPL8	NM_001325777
NtSPL9	KP260636
NtSPL12	NM_001325397
ZmSPL9	EU972995
ZmSPL11	NM_001156062
ZmSPL14	NM_001301523
ZmSPL17	NM_001308478

Supplementary Table S2. Gene specific primers used in this study.

Primer name	Primer sequence
ZmActin-F	5'-CGATTGAGCATGGCATTGTCA-3'
ZmActin-R	5'-CCCACTAGCGTACAACGAA-3'
NtActin-F	5'-TTACGCCCTTCCTCATGCAATT-3'
NtActin-R	5'-GGCGCCACCACCTTGATCTTC-3'
*2*35S-F*	5'-TGACGCACAATCCCACTATC-3'
miR156-R	5'-GCTTTCACCTCTCCTCCACCCTAC-3'
BAR-F	5'-TTGGAGAGGACACGCTGAAATCA-3'
BAR-R	5'-GCTGCCAGAAACCCACGTCAT-3'
GUS-F	5'-GCCGGAATCCATCGCAGCGTA-3'
GUS-R	5'-CCCGCTTCGAAACCAATGCCT-3'
miR156-RT-F	5'-TGCCGTTTTGTTGCTGTTTATCATG-3'
miR156-RT-R	5'- GCCGGAAACAAGCTAAGACAGATG -3'
miR156-realtime-F	5'-GTTTTTGCACTTTGCGTGAA-3'
miR156-realtime-R	5'-GCGATCGATCTCTGTGTCAA-3'
NtSPL2-F	5'-AGGTGTAGCACGTCGCTTTT-3'
NtSPL2-R	5'-AGTTGGGAATGGTTGAGCAC-3'
NtSPL9-F	5'-GAGGTGCATGGCCAAATACT-3'
NtSPL9-R	5'-AAGCAATTTCCGGAAGGAAT-3'
NtAPX-F	5'-GGAGTGGTTGCTGTTGAAGTC-3'
NtAPX-R	5'-GGAGAGCCTTGTCTGATGG -3'
NtCAT-F	5'-CACAGCCACGCTACTCAAGAC-3'
NtCAT-R	5'-CCACCCACCGACGAATAAAG-3'
NtSOD-F	5'-CAACTCCACGGCTTCCAGAC-3'
NtSOD-R	5'-TGGGTCCTGATTAGCAGTGGT-3'
NtCP1-F	5'-GCTATGGAAGGGCTACACCA-3'
NtCP1-R	5'-GCCCTTGTTTTTCAGGATGA-3'
NtCP2-F	5'-CTGAGTTGGACCATGGTGTG-3'
NtCP2-R	5'-GGTAGGATGGTTGCATTGCT-3'
NtCP23-F	5'-GAAAGCTACAGCCGACCAAG-3'
NtCP23-R	5'-TTTACATCCTGCCAATGCAA-3'
NtMC1-F	5'-GCTTCCTCTCCCTCTCCCTA-3'
NtMC1-R	5'-TGGAGGACTCAGGAAAATGG-3'
NtSAG12-F	5'-TACGGAAGGGCTACACCAAC-3'
NtSAG12-R	5'-AGGCCCTTGTTTTTCAGGAT-3'
NtWRKY6-F	5'-CCTTTGCTGGGACAGACAAT-3'
NtWRKY6-R	5'-CACCCTTCCTGAAAGGACAA-3'

NtWRKY53-F	5'-TCGTTTCCCCGAGTAATTTG-3'
NtWRKY53-R	5'-TCTACTGACGAGGCGTGTTG-3'
NtWRKY70-F	5'-AACCCAGTGAAGATGGCAAC-3'
NtWRKY70-R	5'-CGATAATAAGCTCGGGGACA-3'
